# Cerebral Intramural Cells: A Missing Cellular Link Between Vascular Aging and Alzheimer’s Disease

**DOI:** 10.3390/ijms27167353

**Published:** 2026-08-17

**Authors:** Eliza-Mihaela Arbănași, Emil-Marian Arbănași, Tudor-Gabriel Boghițoiu, Stephanie Ada Cărare, Bogdan Andrei Cordoș, Kawthar Braysh, Nicoleta Suciu, Laura Chinezu, Doina Ramona Manu, Axel Montagne, Jennifer Dewing, Eliza Russu, Claudia-Violeta Bănescu, Septimiu-Toader Voidăzan, Roxana-Octavia Cărare

**Affiliations:** 1 Doctoral School of Medicine and Pharmacy, George Emil Palade University of Medicine, Pharmacy, Science and Technology of Targu Mures, 540139 Targu Mures, Romania; arbanasi.eliza@gmail.com (E.-M.A.); tudorboghitoiu@gmail.com (T.-G.B.); exoticvet_umf@yahoo.com (B.A.C.); 2 Centre for Advanced Medical and Pharmaceutical Research (CCAMF), George Emil Palade University of Medicine, Pharmacy, Science and Technology of Targu Mures, 540139 Targu Mures, Romania; laura_chinezu@yahoo.com (L.C.); doina.manu@umfst.ro (D.R.M.); eliza.russu@umfst.ro (E.R.); claudia.banescu@umfst.ro (C.-V.B.); 3 British-Romanian Academic Institute of Neuroscience (BRAIN), George Emil Palade University of Medicine, Pharmacy, Science and Technology of Targu Mures, 540139 Targu Mures, Romania; r.o.carare@soton.ac.uk; 4 Department of Vascular Surgery, George Emil Palade University of Medicine, Pharmacy, Science and Technology of Targu Mures, 540139 Targu Mures, Romania; 5 Clinic of Vascular Surgery, Mures County Emergency Hospital, 540136 Targu Mures, Romania; 6 University Hospital Southampton, Southampton SO16 6YD, UK; stephanie.carare@outlook.com; 7 Dasman Diabetes Institute, Kuwait City 15462, Kuwait; kawthar.braysh@dasmaninstitute.org; 8 European and Research Projects Department, George Emil Palade University of Medicine, Pharmacy, Science and Technology of Targu Mures, 540139 Targu Mures, Romania; nico.suciu03@gmail.com; 9 Department of Histology, George Emil Palade University of Medicine, Pharmacy, Science and Technology of Targu Mures, 540139 Targu Mures, Romania; 10 Institute for Neuroscience and Cardiovascular Research (INCR), Chancellor’s Building, The University of Edinburgh, Little France Crescent, Edinburgh EH16 4SB, UK; axel.montagne@ed.ac.uk; 11 Centre for Clinical Brain Sciences (CCBS), School of Clinical Sciences, College of Medicine and Veterinary Medicine, The University of Edinburgh, Edinburgh EH16 4SB, UK; 12 UK Dementia Research Institute (UK DRI), University of Edinburgh, Edinburgh EH16 4SB, UK; 13 British Heart Foundation–UK Dementia Research Institute (BHF-UK DRI) Centre for Vascular Dementia Research (CVDR), The University of Edinburgh, Edinburgh EH16 4SB, UK; 14 Faculty of Medicine, University of Southampton, Southampton SO17 1BJ, UK; jmd2g08@soton.ac.uk; 15 Department of Genetics, George Emil Palade University of Medicine, Pharmacy, Science and Technology of Targu Mures, 540139 Targu Mures, Romania; 16 Department of Epidemiology, George Emil Palade University of Medicine, Pharmacy, Science and Technology of Targu Mures, 540139 Targu Mures, Romania; septi_26_07@yahoo.com; 17 Faculty of Medicine, George Emil Palade University of Medicine, Pharmacy, Science and Technology of Targu Mures, 540139 Targu Mures, Romania

**Keywords:** cerebral intramural cells, Alzheimer’s disease, smooth muscle cells, intramural periarterial drainage, cerebral amyloid angiopathy

## Abstract

The pathological deposition of amyloid-β (Aβ) in the walls of cerebral blood vessels as cerebral amyloid angiopathy (CAA) is a key feature of Alzheimer’s disease (AD) and is linked to impaired clearance of Aβ via intramural periarterial drainage (IPAD). The spontaneous contractions of cerebral smooth muscle cells (SMCs) are thought to drive IPAD, but the relationship between vascular aging and Aβ accumulation remains unclear. We propose a unified framework centered on cerebral intramural cells (CICs), including arterial SMCs, specialized pericyte subtypes, as mediators of vascular dysfunction and neurodegeneration. We integrate current evidence from studies of cerebral small vessel disease, blood–brain barrier (BBB) dysfunction, pericyte biology, vascular aging, cerebral perfusion, and IPAD. CICs regulate vasomotion, capillary flow, BBB integrity, and IPAD. Their dysfunction impairs perfusion and protein clearance, increasing vulnerability in white matter and the hippocampus. These vascular alterations interact with amyloid and inflammatory processes, contributing to synaptic dysfunction, network disconnection, and neurodegeneration. CICs represent potential therapeutic targets. A CIC-centered framework may help explain the links between vascular dysfunction, impaired Aβ clearance, and neurodegeneration in AD and CAA.

## 1. Introduction

Alzheimer’s disease (AD) is increasingly recognized as a disorder of both the brain parenchyma and cerebral vasculature, in which neuronal proteinopathies (Aβ and tau) intersect with age-dependent failure of cerebral microcirculation, blood–brain barrier (BBB) integrity, and perivascular clearance of fluid and solutes from the brain [1]. AD is the most prevalent cause of dementia, characterized by the deposition of amyloid-β (Aβ) in the walls of blood vessels, cerebral amyloid angiopathy (CAA), and Aβ deposition in the neuropil (neuritic plaques), as well as the buildup of hyperphosphorylated tau in neurons (neurofibrillary tangles, NFTs) [2,3]. These proteinopathies rarely occur in isolation; they unfold against a background of progressive microvascular remodeling in which cerebral microvessels decrease in number, endothelial cells flatten, and smooth muscle cells (SMCs) degenerate, collectively impairing cerebral blood flow (CBF) and cerebrovascular reactivity [1,4]. Resting CBF is reduced and activity-dependent CBF responses are blunted in early AD, and cerebrovascular disease frequently precedes cognitive impairment, indicating a primary rather than secondary role in disease pathophysiology [5,6].

Amyloid precursor protein (APP) represents a transmembrane glycoprotein which produces Aβ peptides after breakdown by beta-site amyloid precursor protein cleaving enzyme 1 (BACE1) and γ-secretase [7]. Subsequent intramembrane proteolysis is mediated by γ-secretase, a multi-subunit enzyme complex in which the catalytic subunits Presenilin 1 (PSEN1) and Presenilin 2 (PSEN2) form the γ-secretase complex. Mutations in these proteins may affect the quantity of Aβ generated and the ratio of degradation products [3]. Furthermore, in people with Down’s syndrome (DS, Trisomy 21), who carry three copies of APP, Aβ42-immunoreactive diffuse deposits are present from the first two decades of life, with ApoE detectable within a subset of these plaques from approximately age 12; N-terminally modified Aβ species accumulate in compacted, neuritic plaques from around age 30 onwards [8].

In this review, we propose a unifying, working hypothesis: age- and disease-driven dysfunction of cerebral intramural cells (CICs)—arterial SMCs, specialized pericyte subtypes, and venular mural cells, together with the basement membranes in which they are embedded—acts as a cellular nexus linking vascular aging to Aβ/tau accumulation, neuroinflammation, and network disconnection. We use CICs as an integrating concept that spans these three populations across microvascular zones, emphasizing their shared positioning “within the wall” (intramural) and their shared roles in vasomotion, BBB maintenance and intramural periarterial drainage (IPAD). This framework is not intended to be exclusive of the other components of the neurovascular unit (NVU) but rather highlight the unique features that intramural cells possess and their contribution to the pathogenesis of cerebrovascular diseases. Through focusing on the biology of CIC, we reconcile three major observations: first, vascular dysfunction precedes clinical symptoms of AD; second, amyloid/tau alone are often insufficient to produce dementia; and third, failures in the clearance and BBB are aligned with aging and small vessel pathology.

## 2. The Clinicopathological Puzzle: Plaques and Tangles Within a Vascularized Brain

AD is defined by neuronal degeneration, extracellular Aβ deposits (plaques), and intracellular tau protein aggregates [9]. Genetic studies indicate that Aβ plays a central role in AD. Mutations in genes that produce Aβ peptides, such as APP, PSEN1, or PSEN2, can lead to autosomal dominant AD [10].

The pathogenesis of AD is complicated, as it requires both Aβ and tau pathology to manifest clinically. AD has two main pathological changes: firstly, senile plaques, which accumulate Aβ peptide, and secondly, NFTs, which contain phosphorylated tau protein [11].

Neuropathologists refer to the existence of just Aβ plaques in the brain as “pathological aging” [12]. Pathological aging frequently exhibits diffuse plaques and is cognitively stable; however, the deposition of Aβ in the walls of cerebral capillaries and arteries as CAA directly correlates with cognitive decline [13]. AD is defined by the interaction and synergistic function among Aβ plaques along with tau proteins, in addition to the cellular response produced by localized glial cells. This leads to neurodegeneration as well as cognitive deficits [14,15]. However, Aβ plaques and phospho-tau NFTs are not exclusive to AD. In instances of hereditary cerebral hemorrhage with amyloidosis of Dutch origin and sporadic cerebral amyloid angiopathy (SCAA), significant β-amyloidosis is observed in the absence of NFTs [16].

Dementia due to AD contributes to approximately 60–90% of all dementia cases, with incidence increasing steeply after age 65 and continuing to rise with advancing age [17]. Apolipoprotein E (APOE) genotype is the strongest common genetic modulator of sporadic AD risk: the early estimate that one or two Apolipoprotein ɛ4 (APOɛ4) alleles raises the likelihood of AD by about 3.5- and, respectively, ~10-fold [18], has since been substantially refined. As reviewed by Jackson et al. [19], APOEε4 effect sizes vary markedly across ethnic groups and are shaped by global and local genetic ancestry and by gene–environment interactions, and possession of APOEε4 significantly increases AD risk while the ε2 allele is protective relative to the most common ε3 allele. Mechanistically, APOEε4 acts through multiple Aβ-dependent and Aβ-independent pathways, including impaired lipid transport, neuroinflammation, glucose metabolism, BBB permeability and cerebrovascular function. The last of these is directly relevant to the CIC framework: APOEε4 activates the cyclophilin A–MMP-9 pathway selectively within pericytes, accelerating mural-cell degeneration and tight-junction breakdown [20,21,22].

These clinicopathological dissociations motivate a permissive-substrate view of disease expression in late life: Aβ and tau act within an aging NVU with declining resilience that determines whether the pathology remains clinically silent or evolves into progressive dementia. We propose that CICs provide the cellular substrate for that resilience, because they simultaneously coordinate perfusion delivery, BBB integrity, basement membrane composition and perivascular clearance, each of which can fail independently of Aβ deposition and independently influence synaptic function and glial activation.

### Evidence That Cerebral Intramural Cell Status, Not Aβ Burden, Separates Pathological Aging from Alzheimer’s Disease

The clinicopathological dissociation between Aβ burden and cognitive status is one of the most robust observations in late-life dementia research. Approximately one third of cognitively unimpaired older adults harbor plaque loads that meet AD neuropathological criteria [12,23], and amyloid-PET-positive cognitively normal individuals can remain stable for many years [24,25,26]. Conversely, individuals with relatively low Aβ burden can develop progressive dementia when accompanied by other pathologies [27]. Therefore, any hypothesis that centers the pathogenesis of AD around Aβ must identify the other biological element that co-determines clinical expression. We describe four essentially separate lines of data that support our claim that this co-determining factor is, to a significant extent, the functional state of CICs.

Firstly, longitudinal clinicopathological cohorts that quantify both parenchymal and vascular Aβ demonstrate that the location of Aβ—within the vessel wall (engaging CICs) versus within the neuropil—separates individuals with similar total Aβ burden into different cognitive trajectories. In the Rush Memory and Aging Project and Religious Orders Study, moderate-to-severe CAA was associated with AD dementia and with decline in perceptual speed and episodic memory independently of plaque burden, tangle burden, infarcts, microinfarcts, and Lewy pathology [28,29]. This constitutes the clearest available within-Aβ comparison: at matched plaque and tangle pathology, the subset with greater intramural Aβ—Aβ in direct contact with SMCs and pericytes—declines faster. A recent extension in 4915 autopsied National Alzheimer’s Coordinating Center individuals further showed that the association of CAA with cognitive impairment operates through modifying the burden and impact of NFTs on cognition, suggesting that wall-intrinsic disease is an upstream amplifier of the traditional AD cascade [30]. The evidence provided by hereditary cerebral hemorrhage with amyloidosis-Dutch type states that dementia develops in the presence of severe vascular Aβ but in the relative absence of neuritic plaques and tangles [31], demonstrating that intramural Aβ engagement of CICs is sufficient to cause cognitive decline on its own.

Secondly, a CIC-injury biomarker predicts cognitive loss independently of tau and Aβ, according to long-term imaging and cerebrospinal fluid (CSF) investigations in living individuals. Nation et al. [32] showed that hippocampal capillary damage and BBB breakdown appear at the earliest stages of cognitive dysfunction independently of Aβ and tau biomarker status by measuring CSF sPDGFRβ, a marker shed by injured capillary pericytes, alongside dynamic contrast-enhanced MRI. This suggests that pericyte injury is a parallel and early contributor to cognitive decline rather than just a downstream consequence of classical AD pathology. Wang et al. [33] quantified this temporal precedence across the lifespan by tracking CSF biomarker trajectories in cognitively unimpaired individuals aged 20–90 years. They discovered that sPDGFRβ starts to rise continuously at age 20, while Aβ42 does not start to decline until roughly age 40. This two-decade gap clearly places pericyte injury upstream of amyloid deposition in the natural history of AD-related pathophysiology. Proteomic profiling of 1670 patients in the BioFINDER cohort identified a signature shared across manifestations of small vessel disease, in which markers of extracellular matrix dysregulation and vascular remodeling—including ELN, POSTN, CCN2 and, most prominently, MMP12—were elevated; these findings were replicated in cerebrospinal fluid from two independent datasets, and a plasma subset outperformed established clinical risk scores in predicting future cerebrovascular events in UK Biobank participants [34].

Thirdly, the same finding is extended to genetic risk by APOE4-stratified research. In a mechanism not observed with APOE2 or APOE3, APOE4 stimulates the CypA–MMP-9 pathway within pericytes, hastening mural-cell degeneration, tight-junction breakdown, and BBB breakdown [20]. This mechanism has been confirmed in human post-mortem tissue, where accelerated pericyte degeneration in AD APOE4 carriers correlates with the magnitude of BBB breakdown and with the accumulation of CypA and MMP-9 in pericytes and endothelial cells, effects that are greater in APOE4 than APOE3 carriers [21]. The principal genetic risk factor for late-onset AD thus acts on the CIC compartment through a wall-intrinsic mechanism, providing a pathway by which genetic risk can dissociate Aβ burden from clinical outcome independently of classical amyloid and tau pathology. In a recent study, APOE4/4 mice were treated with pericytes derived from APOE3/3 human induced pluripotent stem cells and therapeutic effects of apoptotic vesicles (ApoVs) generated from APOE3/3 pericytes were evaluated in APOE4/4 pericytes in vitro [35]. Results demonstrated that pericytes or vesicles derived from pericytes in APOE3/3 may represent a therapeutic strategy for AD in the presence of at least one APOE4 allele.

Finally, time-dependent data-driven analyses of AD place vascular dysregulation among the earliest measurable abnormalities. Iturria-Medina et al. [6] discovered that cerebral vascular dysregulation occurred as an early pathological event and remained more prominent than amyloid, metabolic, or functional measurements across disease stages after evaluating over 7700 brain pictures and tens of CSF and plasma biomarkers from the Alzheimer’s Disease Neuroimaging Initiative (ADNI). Vemuri et al. [5] demonstrated in the Mayo Clinic Study of Aging that vascular and amyloid diseases are statistically independent predictors of cognitive decline in older persons who are cognitively normal, each of which adds to the decline. Together, these data argue against a model in which CIC abnormalities are merely downstream consequences of Aβ and instead support a model in which CIC dysfunction is concurrent with or precedes Aβ deposition, modifying whether equivalent Aβ burdens lead to dementia.

Taken together, the observations above show that (a) within the group of individuals with matched plaque and tangle pathology, those with greater intramural Aβ engagement (CAA) decline faster [28,29]; (b) within the group of individuals with matched Aβ/tau biomarkers, those with higher CSF sPDGFRβ decline faster and show earlier pericyte injury [32,33]; (c) APOE4, the principal genetic risk factor for late-onset AD, acts on the CIC compartment at least through selective activation of the CypA–MMP-9 pathway in pericytes, accelerating BBB breakdown independently of classical amyloid and tau pathology [20,21,22]; and (d) vascular dysregulation is an early, independent contributor to cognitive trajectory in cognitively normal cohorts [5,6]. All four lines of evidence lead to the same conclusion: the clinical manifestation of the Aβ load depends on the functional state of the cerebrovascular intramural compartment.

## 3. Cerebral Intramural Cells: Definition and Biological Scope

### 3.1. Definition

Cerebral CICs comprise arterial SMCs, specialized pericyte subtypes, and venular mural cells, together with the basement membranes, distributed throughout the arterial–capillary–venous tree. Positioned on the abluminal side of endothelial cells, these support vascular integrity and blood flow regulation by generating a range of morphological and transcriptional patterns along the branching vascular tree [36]. This distinction between rapid SMC-mediated and slower pericyte-mediated responses derives predominantly from in vivo two-photon imaging and optogenetic studies in rodents and has not been directly demonstrated in human tissue.

**Figure 1:**
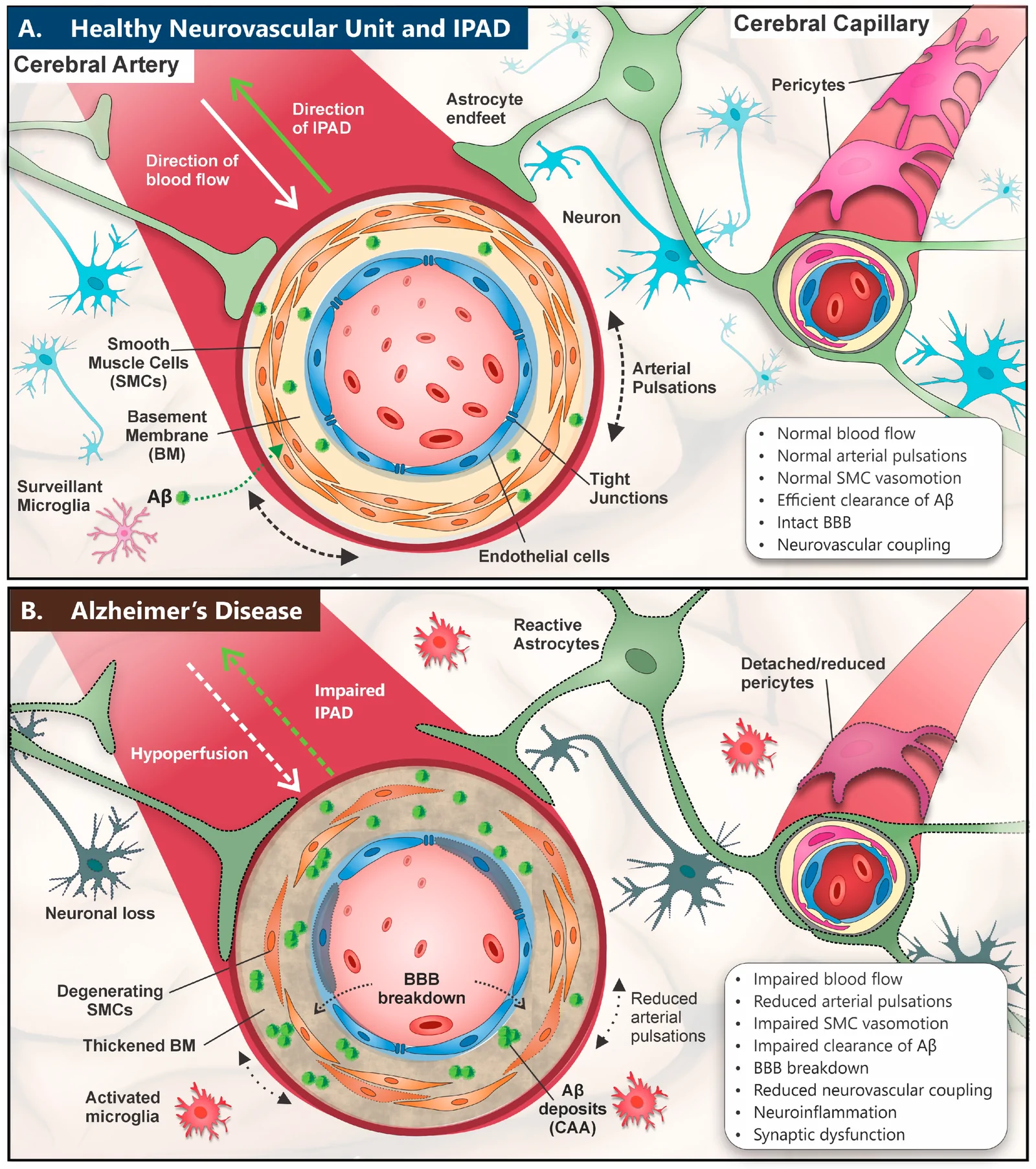
Comparison of the NVU under physiological conditions and in AD ((**A**): in the healthy brain, coordinated interactions between endothelial cells, VSMCs, pericytes, astrocytic endfeet, neurons, and microglia maintain CBF, BBB integrity, and neurovascular coupling. IPAD, driven by arterial pulsations and VSMC vasomotion, facilitates the clearance of interstitial Aβ along basement membranes in the opposite direction to blood flow; (**B**): in AD, impaired IPAD promotes vascular Aβ accumulation, leading to CAA, degeneration of mural cells, basement membrane thickening, reduced arterial pulsatility, BBB disruption, chronic neuroinflammation, impaired neurovascular coupling, and progressive neuronal dysfunction). Figure created with CorelDRAW Graphics Suite 2025 (Alludo, Ottawa, ON, Canada).

The NVU, as defined and subsequently revised [37,38,39], is a functional construct that intentionally spans the parenchymal–vascular interface, integrating neurons, astrocytes, microglia, endothelial cells, and mural cells around the coordinated regulation of CBF and BBB homeostasis. The CIC concept is narrower and complementary: it isolates those cells anatomically embedded within the vessel wall and its basement membranes that share a unifying mechanical identity, namely the generation, transmission, or modulation of the contractile and pulsatile activity that drives vasomotion, neurovascular coupling (NVC) along resistance and capillary segments, and IPAD. Endothelial cells and astrocytes, although indispensable members of the NVU, are not intramural in this strict sense: endothelial cells constitute the luminal interface, whereas astrocytic endfeet contact the abluminal basement membrane from the parenchymal side (Figure 1).

Restricting the CIC framework to cells located inside the wall generates three predictions not foregrounded by the NVU perspective. First, vasomotion-driven IPAD failure is a primary, wall-intrinsic clearance defect that is mechanistically different from glymphatic CSF inflow deficits (which rely on AQP4 polarization at astrocytic endfeet) and BBB transcytosis impairment (an endothelial process) [40,41,42,43,44]. Second, age- and amyloid-related dysfunction manifests as a graded, zone-specific phenotype along the arterio–capillary–venous axis, moving from pial SMCs to ensheathing pericytes and capillary pericytes to venular mural cells, a pattern supported by single-cell transcriptomic zonation studies but obscured when the NVU is treated as a single functional unit [36,45]. Third, the framework identifies wall-intrinsic processes—SMC contractile loss, pericyte phenotypic drift, and basement-membrane remodeling—as therapeutic targets largely orthogonal to the endothelial and astrocytic targets that are currently predominating in the NVU literature [46,47,48].

The CIC concept is not intended to replace established nomenclature (SMCs, pericytes, venular mural cells) but rather to provide an organizing framework that highlights a shared intramural location and a shared set of wall-dependent functions.

High quantities of α-smooth muscle actin are expressed by SMCs, which encircle pial arteries and penetrating arterioles. This allows for quick modulation of CBF and vascular diameter. Ensheathing pericytes, which similarly express α-smooth muscle actin and control vascular tone, envelop arteries in the arteriole–capillary transition (ACT) zone. However, the contractile dynamics of pericytes are slower compared to those of SMCs [49].

### 3.2. Shared Functions Relevant to Alzheimer’s Disease

Across cerebral areas, CICs contribute to four interdependent processes that are repeatedly implicated in AD: vasomotor control and vasomotion, NVC and conducted vascular responses, BBB induction and maintenance through endothelial cross-talk, and IPAD through regulation of basement membrane structure and vessel wall dynamics. This framing emphasizes that “hypoperfusion”, “BBB breakdown”, and “clearance failure” can be manifestations of a single, spatially distributed cellular failure mode derived from the vessel wall.

Through interfering with CBF, perfusion, and BBB integrity, dysfunction of the cerebral microvasculature leads to neurological illness [50,51]. Across vascular zones, these changes include pathological remodeling and degeneration of mural cell populations [52]. Such microvascular pathology is becoming more widely acknowledged as a major cause of dementia, especially in AD, where dysfunctional mural cells may be a major factor in the course of the illness.

Mechanistic integration across microvascular zones (pial arteries → penetrating arterioles → ACT → capillaries → venules) centered on CIC is required for a better understanding of pathophysiological processes and targeted therapies, rather than treating “BBB”, “hypoperfusion”, and “clearance failure” as separate phenomena. Since CICs are zone-specific [36], AD-relevant phenotypes may emerge in specific zones (e.g., ACT ensheathing pericytes controlling upstream resistance vs. capillary pericytes shaping flow heterogeneity) [53].

## 4. Vascular Contributions to Cognitive Impairment and Dementia

Vascular contributions to cognitive impairment and dementia (VCID) describes the spectrum of cognitive impairment arising from vascular brain injury, ranging from post-stroke cognitive impairment to vascular dementia and to mixed AD–vascular dementia [54,55]. VCID and AD are mechanistically interrelated and frequently coexist: in most post-mortem instances, CAA co-occurs with AD neuropathological changes, and the two pathologies appear to reinforce each other through a common impairment of Aβ clearance [56,57]. CAA is highly prevalent in AD, being identified in approximately 79% of autopsy-confirmed AD cases, although its severity varies considerably, ranging from mild focal vascular amyloid deposition to advanced CAA associated with clinically significant cerebrovascular complications [58]. VCID is closely tied to cerebral small vessel disease (cSVD), since the majority of pure vascular dementia cases in the absence of large-vessel stroke arise from accumulating cSVD-related brain injury, which collectively accounts for more than 20% of all dementias globally [59,60]. Collagen 4 (COL4)-positive pericytes in the frontal deep white matter are significantly decreased in normal aging, as well as across different dementias including AD, mixed AD vascular dementia (VaD) as well as post-stroke dementia (PSD) and VaD [61,62].

### 4.1. Cerebral Amyloid Angiopathy

CAA is defined neuropathologically by the deposition of Aβ within the walls of cortical and leptomeningeal small arteries, arterioles and capillaries; venous involvement is occasionally reported but is not a defining feature [57,63]. Unlike the Aβ42-dominated parenchymal plaques, vascular Aβ deposits are enriched in the Aβ40 species, which builds up within the IPAD pathways, suggesting thus different mechanisms of deposition for vascular versus parenchymal amyloid [64,65]. Deposition begins in the outer basement membrane and progresses into the tunica media, with eventual fibrinoid necrosis, SMC loss, microaneurysm formation and lobar hemorrhage [66,67]. Two subtypes are recognized: CAA-type 1, in which Aβ extends into cortical capillaries, is strongly associated with APOEε4 and with capillary occlusion and CBF disturbances, and CAA-type 2, in which capillaries are spared, and it is not associated with APOEε4 [64,65,68]. The Boston criteria v2.0 [69] provide the current clinical diagnostic standard, incorporating MRI markers such as lobar microbleeds, cortical superficial siderosis, severe perivascular space enlargement in the centrum semiovale, and a multispot pattern of white matter hyperintensities, with superior sensitivity over the prior criteria without loss of specificity. Within the CIC framework, CAA represents the prototypical wall-intrinsic disease: vascular Aβ directly engages SMCs and capillary pericytes, impairs their contractile function, and creates a self-reinforcing cycle in which progressive IPAD failure drives further vascular Aβ accumulation, accelerating both CAA and AD pathology [57].

### 4.2. Cerebral Small Vessel Disease

**Figure 2:**
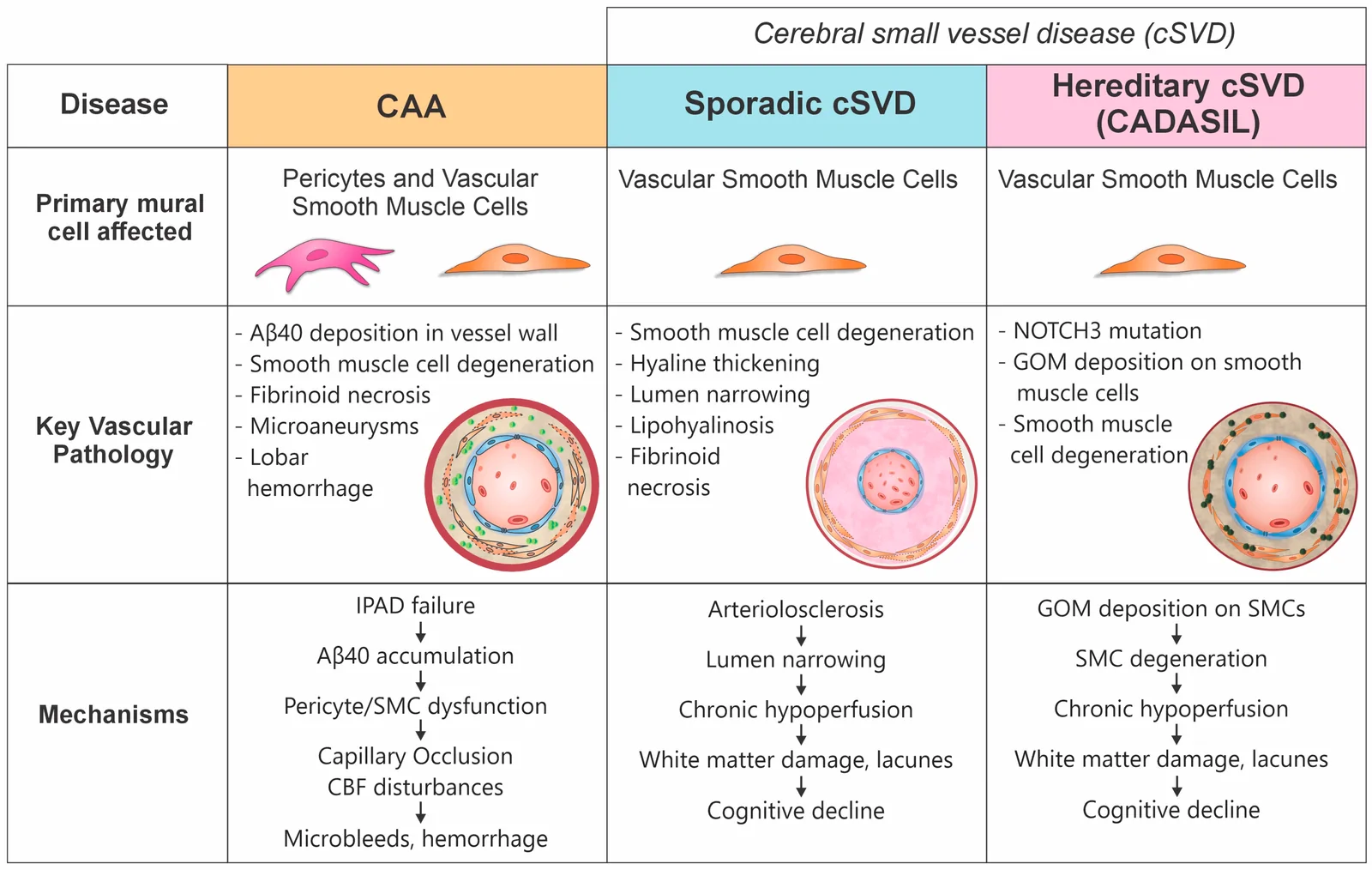
Comparison of the principal mural cell populations, vascular pathology, and pathogenic mechanisms in CAA, sporadic cSVD, and hereditary cSVD (CADASIL). Figure created with CorelDRAW Graphics Suite 2025 (Alludo, Ottawa, ON, Canada).

cSVD is an umbrella term for a heterogeneous group of conditions affecting the small perforating arteries, arterioles, capillaries, and venules of the brain [59,60]. The principal sporadic forms are age- and hypertension-related arteriolosclerosis, characterized by SMC degeneration, hyaline thickening, lipohyalinosis and, in more severe cases, fibrinoid necrosis. The principal genetic form is cerebral autosomal-dominant arteriopathy with subcortical infarcts and leukoencephalopathy (CADASIL), caused by *NOTCH3* mutations that lead to extracellular accumulation of granular osmiophilic material (GOM) on the surface of SMCs and to progressive SMC degeneration [60]. The STRIVE-2 consensus [70] standardizes the imaging biomarkers of cSVD: white matter hyperintensities, recent small subcortical infarcts, lacunes, cerebral microbleeds, enlarged perivascular spaces and brain atrophy [54,60]. The shared mural-cell pathology across CAA, sporadic arteriolosclerosis, and CADASIL is one of the principal motivations for the CIC framework: having different aetiologies, each condition primarily damages a defined subset of intramural cells, and it is this convergence on the vessel wall that the CIC concept is designed to capture [54,60,71]. The principal mural-cell populations, vascular lesions, and pathogenic mechanisms distinguishing CAA, sporadic cSVD, and CADASIL are summarized in Figure 2.

## 5. Anatomical Organization of the Cerebral Microvasculature and CIC Subtypes Along the Vascular Tree

### 5.1. Macro-to-Micro Vascular Organization

Although regional variations are still poorly understood, the cerebral microvascular network is arranged into discrete anatomical and functional zones, best characterized in the cerebral cortex [72]. The CBF is supplied and drained by the interconnected two-dimensional networks of pial arteries and veins on the surface of the brain. The middle, anterior, and posterior cerebral arteries are the sources of these pial vessels, which are joined by leptomeningeal collaterals that permit blood redistribution between arterial areas [72]. The superior sagittal sinus is one of the main venous sinuses into which pial venules drain blood [73].

Permeating arterioles and venules descend into the cerebral cortex from the pial vascular plexus to deliver and respectively drain blood from the deeper layers of tissue [73,74]. Red blood cells slow down in relation to arterioles but remain faster than in capillaries in the ACT zone, which is created by these penetrating arterioles and is usually described as the first four branch orders [45]. Capillaries are the main location for the exchange of nutrients and oxygen and have the largest surface area in the cerebral vasculature [75]. Blood flows from capillaries towards the pial veins via ascending and postcapillary venules [76].

### 5.2. Cerebral Intramural Cell Subtypes Along the Vascular Tree

**Figure 3:**
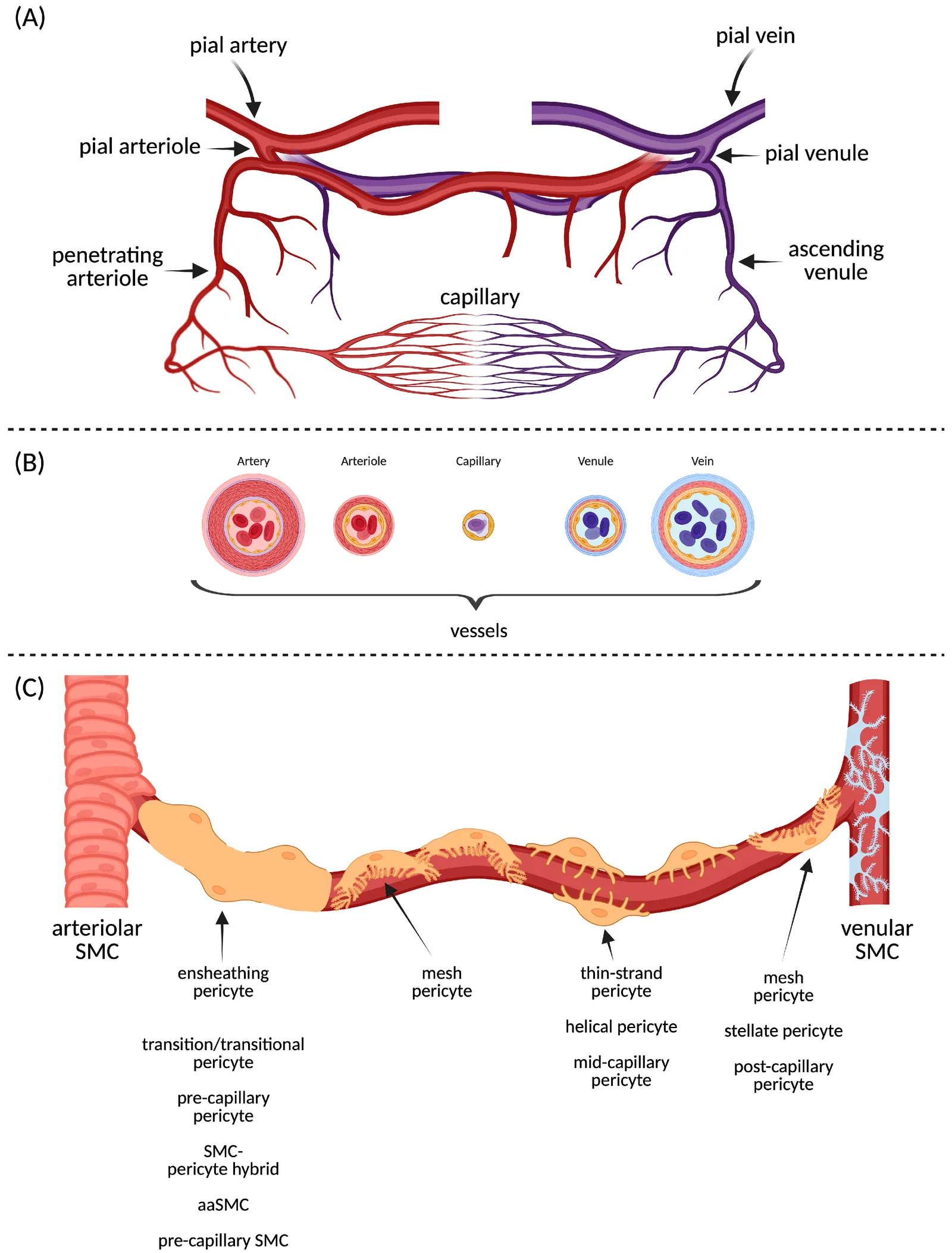
Anatomical organization of the cerebral microvasculature and pericyte morphology along the vascular tree ((**A**): schematic representation of cortical microcirculation showing blood flow from pial arteries through pial arterioles and penetrating arterioles into the capillary bed, followed by drainage through ascending venules, pial venules, and pial veins; (**B**): cross-sectional comparison of vessel types along the vascular tree, illustrating structural differences between arteries, arterioles, capillaries, venules, and veins, including variations in vessel diameter, wall thickness, and cellular composition; (**C**): distribution and morphological diversity of pericytes along the microvascular network. Arteriolar SMCs transition into ensheathing pericytes and pre-capillary pericytes, followed by mesh, thin-strand, and helical pericytes in mid-capillaries, and stellate/post-capillary pericytes toward the venular side, reflecting gradual changes in contractile properties and vascular support). Figure adapted from Uemura et al. [88] and created in BioRender. Arbanasi, E. M. (2026) https://BioRender.com/sqnzns7.

Mesh and thin-strand pericytes represent the two primary types of pericytes that encircle capillaries in the brain; mesh pericytes are found nearer the ACT zone [75] (Figure 3). While mesh pericytes have more intricate branching, both pericyte types have the distinctive “bump-on-a-log” shape, which consists of a spherical soma and elongated processes. Together, these processes cover about 90% of the capillary length in the mouse cortex, extending longitudinally along capillaries and making contact with nearby pericytes [77].

Pericytes create unique peg-and-socket connections with endothelial cells after becoming entrenched in the capillary basement membrane [78]. Capillary pericytes have low or undetectable amounts of α-smooth muscle actin, indicating poor contractile capacity, different from ensheathing pericytes and SMCs. They can, however, control capillary diameter and circulation in response to vasoconstrictive stimuli and variations in intramural pressure [49,79,80]. They also retain some of the contractile machinery inherent in mural cells [81]. These cells are believed to play a major role in slower aspects of CBF control, such as delayed stages of the hemodynamic response and basal capillary tone [45,82].

Mesh pericytes surround postcapillary venules similarly, but it is uncertain if they constitute a separate population and their functional significance is still unclear [49]. Venular SMCs, that exhibit stellate shape, reduced α-smooth muscle actin expression, and low contractility, encircle ascending venules, in contrast to the arteriolar SMC phenotype [83]. Their functions in maintaining vascular homeostasis remain mainly unclear.

Recent single-cell and spatial transcriptomic studies have highlighted remarkable vascular zonation and endothelial–mural cell interactions across the vascular tree [84]. They provide strong support for the working hypothesis that vascular dysfunction is heterogeneous and zone-specific. A cation-transporting ATPase 13A5 (*Atp13a5*) marker was identified through single-cell transcriptomics as specific to brain pericyte lineage, leading to the development of an inducible *Atp13a5-CreER* knock-in model with tdTomato reporter expression that reliably labels central nervous system (CNS) pericytes while sparing peripheral organs [85]. This model represents a valuable tool to study the functions of pericytes in health and disease. Furthermore, the hypothesis that forces generated by the cerebral vascular SMCs (VSMCs) drive IPAD through deformation of the vascular basement membrane has previously been proposed, mathematically modeled, and experimentally demonstrated [86,87]. Together, these advances provide an opportunity to directly test the CIC framework by combining selective pericyte manipulation using the *Atp13a5-CreER* model with quantitative assessment of IPAD—for example through the clearance of soluble fluorescent dextrans. Such experimental approaches may help define the specific contribution of pericytes to IPAD and clarify how dysfunction of distinct CIC populations contributes to impaired vascular clearance during aging and Alzheimer’s disease [85].

Cerebral autosomal dominant arteriopathy with subcortical infarcts and leukoencephalopathy (CADASIL) and cerebral autosomal recessive arteriopathy with subcortical infarcts and leukoencephalopathy (CARASIL) are two of the hereditary small vessel diseases accompanied by degeneration of mural cells [88].

## 6. Intramural Cells and the Blood–Brain Barrier

The BBB is a highly specialized structure constructed of brain endothelial cells (BECs) connected by tight junction proteins, such as occludins, claudins, and zonula occludens proteins ZO-1, ZO-2, and ZO-3. Pericytes, astrocytic endfeet, and a continuous basement membrane surround the BECs [52]. Through mutual signaling that modifies endothelial development and barrier tone, pericytes and BECs, which share the basement membrane, work together to control vascular permeability. Astrocytic endfeet adhere to the abluminal basement membrane and contribute to barrier maintenance, while microglia, the resident immune cells of the CNS parenchyma, surveil the perivascular space and respond to signals of injury and neuroinflammation [89]. A damaging loop of neuroinflammation, synaptic dysfunction, and progressive neuronal damage may be started and maintained by BBB malfunction, which can result from poor molecular transport, aberrant angiogenesis, vascular degeneration, or cerebral hypoperfusion [90].

Converging evidence from animal models, post-mortem tissue, neuroimaging, and CSF biomarker studies demonstrates BBB breakdown as a consistent and early characteristic of AD [91,92]. Through endothelial tight junctions, specialized transporters, inhibited transcytosis, and pericyte and astrocyte support, the BBB controls the movement of chemicals and cells between the blood and the brain [52]. Hippocampal BBB leakage is associated with cognitive deterioration from the earliest stages of the disease, and BBB failure allows immune cells and serum proteins to enter the brain, often near CAA lesions [32,93].

This mechanism is strongly related to pericyte injury: loss of pericytes is associated with decreased claudin-5 and other tight junction proteins, which promotes Aβ accumulation and neuronal damage [33,93]. Pericyte integrity is essential for both vascular and neuronal homeostasis, as demonstrated by experimental pericyte ablation, which results in acute BBB disruption, a significant decrease in CBF, and rapid neuronal loss mediated in part by depletion of pericyte-derived pleiotrophin, a neurotrophic factor [94].

**Figure 4:**
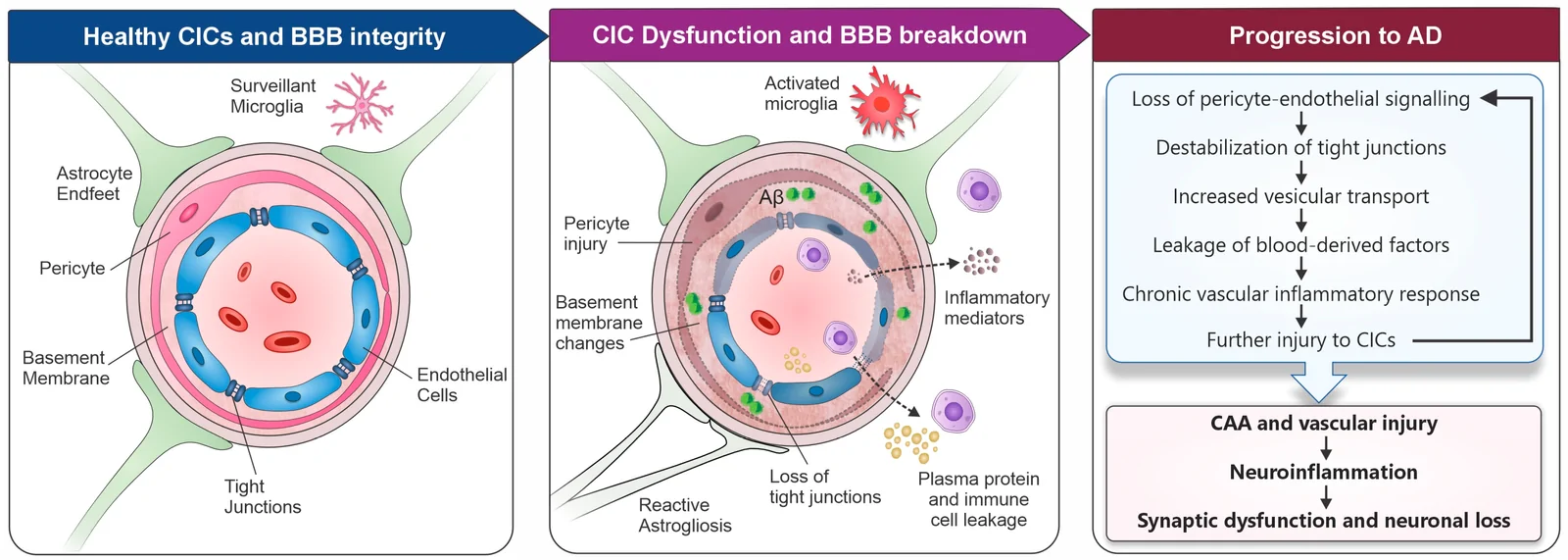
Contribution of CICs to BBB maintenance and AD pathology. ((**Left**): under physiological conditions, coordinated interactions between pericytes, endothelial cells, astrocytic endfeet, basement membrane, and surveillant microglia preserve BBB integrity and vascular homeostasis; (**Middle**): CIC dysfunction results in pericyte injury, basement membrane remodeling, Aβ accumulation, tight junction disruption, inflammatory mediator release, reactive astrogliosis, and leakage of plasma proteins and immune cells across the BBB; (**Right**): progressive loss of pericyte–endothelial signaling initiates a self-amplifying cascade of BBB breakdown, vascular inflammation, CAA, synaptic dysfunction, and neuronal loss, ultimately contributing to AD progression). Figure created with CorelDRAW Graphics Suite 2025 (Alludo, Ottawa, ON, Canada).

Therefore, BBB disintegration is both initiated and amplified by CIC dysfunction. Loss of pericyte–endothelial signaling destabilizes tight junction architecture and increases vesicular transport, while leakage of plasma proteins and inflammatory mediators into the perivascular space creates a hostile biochemical environment that further stresses intramural contractile cells. As a result, BBB degradation turns into a wall-centered feed-forward mechanism that links vascular aging to progressive cognitive decline and hippocampal susceptibility (Figure 4).

## 7. Perfusion Control by Intramural Cells: Neurovascular Coupling and Capillary Flow Quality

The leptomeningeal arteries are the primary source of vascular resistance [95]. To optimize perfusion, dilatation of intracerebral vessels at the point of activation should be accompanied by dilation of the upstream leptomeningeal arteries [96]. Activated areas require further vascular modifications, whereas inactive areas are vascularized by other branches of the pial artery.

Brain activity regulates CBF through coordinated interactions between neurons, glia, and vascular cells. Neurons and glia initiate vasodilation signals, but endothelial cells, pericytes, and SMCs work together to organize vascular changes which raise CBF in the triggered area and correlate with the period of activation. Pathological diseases, including hypertension, AD, and ischemic stroke, alter neurovascular coupling. As a result, CBF no longer matches the metabolic requirements of the tissue. Reactive oxygen species negatively affect cerebral blood vessels, contributing to cerebrovascular dysregulation [97]. NADPH oxidase is a family of enzymes that contributes significantly to cerebral vascular radicals in hypertension and AD models [98,99].

The role of pericytes in controlling capillary blood flow has been studied for a long time now. Capillaries dilate before arterioles during NVC and constrict during ischemia [37]. It has also been shown that both congenital and adult-onset pericyte loss result in impaired NVC and decreased CBF [94]. Inconsistent vascular nomenclature and the challenge of distinguishing between normal and pathological consequences in models with broad pericyte depletion are the main causes of these disparities [45].

Capillary pericytes actively control basal capillary tone, as evidenced by recent optogenetic and ablation studies [49]. Their loss results in local dilatation and increased blood flow, while their stimulation causes slow capillary constriction and decreased flow [49,77]. Disrupted capillary flow has been noted in AD patients and mouse models [100], and pericyte loss increases capillary flow heterogeneity, lowering oxygen extraction efficiency [101,102].

A key factor in cerebrovascular regulation is represented by nitric oxide (NO). As established by Garthwaite and colleagues, NO generated by neuronal and endothelial NO synthase diffuses freely across cell membranes and acts as a volume transmitter, reaching all targets within a diffusion radius rather than a defined partner cell; its effective signaling range is set by the balance between the rate of synthesis and the rate of inactivation, and its principal receptor, soluble guanylyl cyclase, responds to NO concentrations in the low nanomolar range and below [103]. Two consequences follow for the CIC framework. First, because NO acts on all targets within its diffusion radius rather than on a designated partner cell, a single NO source can act simultaneously on endothelial cells, pericytes and SMCs, providing a mechanism by which contractile activity may be synchronized along a vascular segment. Second, conditions that increase NO consumption—oxidative stress, NADPH oxidase-derived superoxide, and hemoglobin exposure following microhaemorrhage—curtail NO signaling even when synthase activity is preserved, and would therefore be expected to impair vasomotion and conducted vasodilation without any loss of the enzymatic source.

Reduced cerebral perfusion can lead to ischemic lesions that exacerbate dementia [1]. Insufficient CBF can affect trafficking across the BBB for Aβ [90]. Reduced CBF may impede Aβ clearance and therefore increase its buildup in the brain. Furthermore, reducing CBF may hinder the production of brain proteins, which is crucial for cognitive function [1]. Alterations to the microvasculature in AD may contribute to the brain dysfunction that causes dementia.

Abrupt pericyte loss precedes endothelial activation in human cSVD [104], and experimental pericyte depletion induces endothelial inflammatory and interferon-associated responses, alters vascular homeostasis, impairs cerebral blood flow regulation, and promotes endothelial phenotypic transitions that may participate in vascular repair and pericyte repopulation [105]. These findings support the hypothesis that mural cell dysfunction should be considered within a broader framework of dynamic endothelial–mural cell interactions rather than as an isolated process. Vascular dysfunction, BBB breakdown, impaired clearance pathways, and neurogliovascular unit alterations contribute to cognitive decline and dementia [106].

Taken together, the evidence above suggests that perfusion failure in AD begins as impaired flow regulation rather than a reduction in resting CBF. Early dysfunction of the CIC coupled with endothelial cell dysfunction can disrupt conducted vasodilation in upstream arterioles and increase the variability of capillary transit-time, lowering oxygen extraction efficiency before any measurable vessel loss. These microhemodynamic changes are detectable by transit-time imaging and may be one of the earliest measurable consequences of CIC aging relevant to cognitive decline.

## 8. White Matter Vulnerability as a Readout of Intramural Dysfunction

One of the main characteristics of AD is white matter damage that disrupts the connectivity of brain networks, which leads to cognitive loss [107,108]. Low capillary density and distance from arterial supply make white matter particularly susceptible to hypoperfusion, which worsens with age as a result of increased capillary loss, decreased proangiogenic signaling, and the development of regressed “string vessels” [109]. White matter pathology is exacerbated by arteriole tortuosity and venous collagenosis in aging and AD, which also limit glymphatic CSF outflow, encourage BBB leakage, edema, and worsen blood flow [110].

White matter is therefore a low-reserve compartment in which modest CIC dysfunction can produce measurable tissue injury. Progressive impairment of arteriolar tone, increased capillary flow heterogeneity, and venous outflow restrictions together promote chronic oligemia, edema, and impaired perivascular clearance. This pattern is consistent with the observation that white matter hyperintensities track with cSVD imaging burden [60] and contribute to cognitive decline at a stage when cortical neurodegeneration may still be subtle.

## 9. Intramural Cells at the Interface with Amyloid and Tau Biology

The amyloid cascade hypothesis (ACH) proposes that Aβ plaques are the primary cause of AD pathogenesis, based on genetic evidence [111]. Over-production of Aβ and/or lack of Aβ clearance in sporadic AD leads to the progressive development of Aβ oligomers, which deposit into Aβ plaques [111]. Aβ oligomers and plaques lead to hyperphosphorylation of tau, accelerating neuronal and synaptic dysfunction [111]. Recent setbacks in clinical studies of anti-Aβ antibodies cast the linearity of the ACH under uncertainty [111].

CAA is a form of small vessel disease and consists of the deposition of Aβ in the walls of leptomeningeal arteries and intracerebral capillaries and arterioles [60,112]. The pattern of distribution of Aβ in CAA suggests that it represents a failure of intramural periarterial drainage of interstitial fluid and Aβ [113,114]. Mathematical and experimental studies suggest that the motive force for IPAD is represented by the spontaneous contractions of CIC, a process known as vasomotion [115]. Loss of vasomotion precedes the development of CAA in experimental models [86].

A systematic review and meta-analysis of neuropathological series established the prevalence of CAA across populations. Among older individuals representative of the general population, mild-to-severe CAA was present in 41.5% and moderate-to-severe CAA in 23.0%; among individuals with AD the corresponding figures were 79.2% and 47.5% [58]. Sporadic CAA is therefore present to some degree in the large majority of AD brains and is moderate or severe in approximately half, which establishes that the CIC–CAA–AD link described here applies to most, rather than a minority, of AD cases. The familial forms follow a different distribution. In hereditary cerebral hemorrhage with amyloidosis of Dutch type and the related *APP* mutations affecting the Aβ mid-region, CAA is essentially universal, severe, and present from early adult life, while parenchymal plaque and tangle pathology may be comparatively sparse [16,31].

In AD animal models such as APP/PS1 and 5xFAD, Aβ also encourages capillary flow pausing and regression, which worsens brain perfusion [116]. Furthermore, prior to major pericyte loss, Aβ can cause pathological pericyte constriction, which lowers capillary blood flow [117]. In both human [118,119,120] and animal models [121,122,123], decreased CBF is strongly linked to cognitive decline and increased Aβ deposition. These findings suggest that pericyte dysfunction and loss contribute to impaired microcirculation, disruption of the BBB, and cognitive deficits in AD, although the exact timeframe for the pericyte loss and CBF decline to occur is unclear.

Aβ can be considered not only a parenchymal stressor but also a vasoactive and wall-active agent that perturbs CIC tone and microvascular stability. Early oligomeric Aβ can drive pericyte-mediated capillary constriction [117], promote capillary stalling and regression [124], and thereby worsen perfusion and oxygen extraction. These changes are predicted to increase Aβ retention by slowing clearance across the BBB, reinforcing a self-amplifying loop between intramural dysfunction and amyloid accumulation.

While Aβ builds up in the extracellular spaces of the brain, in AD there are intracellular modifications of tau that becomes hyperphosphorylated in the form of neurofibrillary tangles and spreads across different areas of the brain with progression of disease severity [125]. When primary human VSMCs were treated with mutant tauP301L, the VSMCs showed dramatic changes in their cytoskeleton morphology characterized by the formation of dendritic-like phenotype (lamellipodium) and loss of polarized cytoskeleton microfilaments (F-actin) which associated with increased tau [51]. Immunoblot analysis of total lysates from the human VSMCs showed that dysfunctional VSMCs can produce high levels of both total and phosphorylated tau [51]. In post-mortem human brains with AD, small and medium-sized arteries showed early significant loss of α-SMA immunostaining present at Braak stage I and II–III, along with accumulation of phosphorylated paired helical filament tau in the perivascular space of the intraparenchymal vessels [126].

## 10. Intramural Cells, Basement Membranes, and Clearance of Brain Waste

Aβ, along with other metabolites, builds up in the brain parenchyma in AD due to their impaired clearance from the brain parenchyma [127]. Two main routes of clearance are proposed: IPAD, where waste is cleared along the arterial basement membrane in the opposite direction of blood flow [87,128], and the glymphatic system, where CSF enters through astrocytic Aquaporin-4 channels and drains towards the surface of the brain along venous perivascular spaces [129]. This, however, does not explain why CAA is most frequent in cerebral capillary and arterial walls, rarely observed in the walls of veins [63]. CSF enters the brain through the perivascular spaces surrounding arterioles and mixes with interstitial fluid.

Changes in the vascular basement membrane and perivascular gaps, which are frequently seen in aging and AD, are believed to hinder brain clearance, but the exact anatomy is yet unknown [87]. Reduced vasomotion or SMC loss may consequently encourage vascular Aβ buildup, as shown in CAA, since both systems depend on the pulsatile activity of arteriolar SMCs to drive CSF–interstitial fluid exchange [130]. Furthermore, waste clearance is further compromised by the disruption of CSF–interstitial fluid dynamics caused by perivascular space enlargement, which happens with aging and small vessel illness [131].

Aβ is cleared across the BBB through P-glycoprotein efflux via the luminal portion and low-density lipoprotein receptor-related protein 1 (LRP-1)-mediated uptake on the abluminal portion. In AD models, loss of endothelial LRP-1 speeds up Aβ accumulation and cognitive decline [132,133]. As per the findings of Bell et al. [20], Montagne et al. [22], and Halliday et al. [21], pericytes also eliminate Aβ through LRP1–APOE mechanisms. However, elevated Aβ levels or the APOE4 isoform hinder pericyte function, slow Aβ transport via VLDLR, and promote BBB damage through cyclophilin A–MMP9 pathways, all of which contribute to pericyte loss and the advancement of AD.

The buildup of Aβ, loss of mural cells and the changed gene expression in NVU are strongly associated with microvascular dysfunction, which is becoming more widely acknowledged as a factor in cognitive impairment. Aβ accumulation, neurodegeneration, and inflammation are made worse by these alterations, which also impair CBF, neurovascular coupling, BBB integrity, waste clearance, and vascular basement membrane composition [83].

**Figure 5:**
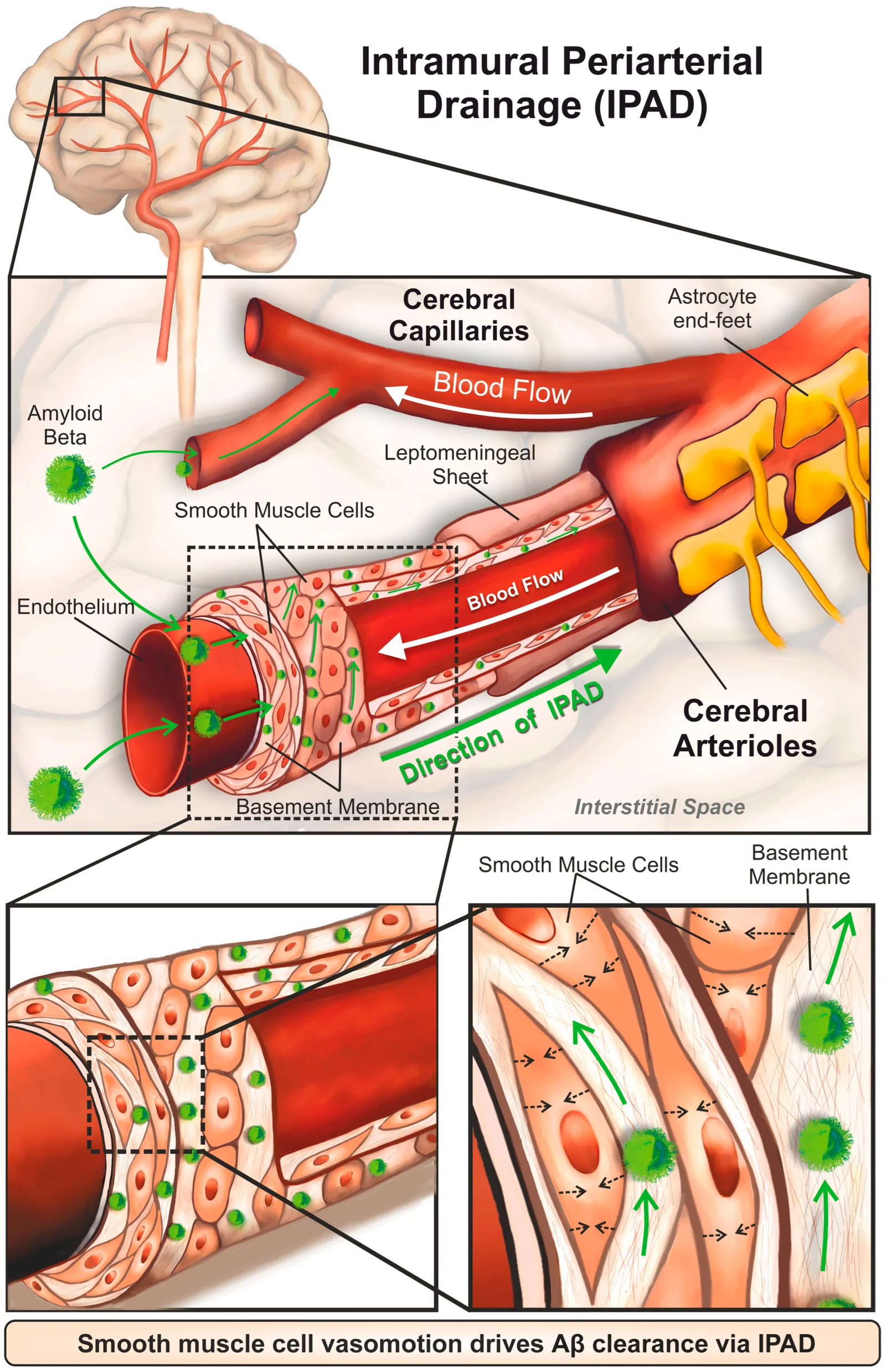
Schematic representation of the IPAD pathway responsible for the elimination of interstitial solutes from the brain. Aβ produced within the brain parenchyma enters basement membranes surrounding capillaries and cerebral arterioles, where it is transported along vascular smooth muscle cell basement membranes in the opposite direction to blood flow. IPAD is driven by vascular smooth muscle cell vasomotion and arterial pulsations, enabling efficient clearance of soluble Aβ toward leptomeningeal and extracranial drainage pathways. Impairment of this clearance mechanism promotes vascular Aβ retention and contributes to cerebral amyloid angiopathy and AD. Figure created with CorelDRAW Graphics Suite 2025 (Alludo, Ottawa, ON, Canada).

Since IPAD depends on arteriolar pulsatility and vasomotion [87,128], CIC dysfunction directly limits Aβ clearance along this route (Figure 5). SMC loss, phenotypic drift of pericytes, and age-dependent remodeling of the basement membrane narrow the intramural drainage path and favor Aβ retention within the vessel wall. Enlargement of perivascular spaces, seen with aging and cSVD [129] further disrupts CSF–interstitial fluid exchange. This combination helps explain why CAA preferentially affects arterial walls and why Aβ accumulates even when its production is stable.

## 11. Neuroinflammation as an Amplifier of Intramural Pathology

Over the past decade, neuroinflammation has emerged as an integral, rather than incidental, component of AD pathophysiology. Multiple lines of evidence indicate that the Aβ deposition and NFT are accompanied by sustained activation of innate immune signaling within the CNS, and that complement activation and microglial synaptic pruning can precede measurable parenchymal proteinopathy and contribute to early synaptic dysfunction [134]. Microglial metabolic reprogramming toward glycolysis further sustains and amplifies this inflammatory state in established disease [135]. Within the CIC framework, however, the salient point is where this inflammatory activity is concentrated and what it acts upon. A growing body of work places the most intense and persistent inflammatory signaling at the perivascular interface—precisely the anatomical compartment occupied by SMCs, pericytes, and venular mural cells. Neuroinflammation in AD is therefore not only a parenchymal phenomenon but, to a substantial extent, an intramural and perivascular event, with CICs serving as both targets and contributors.

This perivascular concentration of inflammation is exemplified by SCAA, in which extracellular Aβ—predominantly Aβ1-40—accumulates within leptomeningeal and cortical vessel walls. The substantial clinical and pathological overlap between CAA and AD, and the shared dependence on impaired perivascular Aβ drainage in both diseases [55,56,57], reinforce the conclusion that CIC degeneration and neuroinflammation are mechanistically inseparable rather than parallel processes.

At the cellular level, the integration of neuroinflammation with intramural pathology is anatomically explicit: vascular Aβ deposition is consistently flanked by perivascular microglial activation and reactive astrocytosis, spatially concentrated around amyloid-laden vessels and centered on the mural cell layer [65]. Activated perivascular glia release pro-inflammatory cytokines, matrix metalloproteinases, and reactive oxygen and nitrogen species directly into the perivascular space, where they impair endothelial nitric oxide signaling, disrupt PDGFRβ-dependent pericyte survival signaling, and accelerate VSMC contractile dedifferentiation [136,137,138]. These same mediators degrade the LRP-dependent transcytotic clearance machinery at the abluminal endothelial surface [139], and obstruction of periarterial drainage by vascular Aβ then perpetuates local cytokine retention and immune activation [40,57]. The result is a self-reinforcing pathogenic loop in which CIC degeneration impairs clearance, retained Aβ amplifies perivascular inflammation, and inflammation further accelerates CIC loss—positioning neuroinflammation not as an independent process but as a quantitative amplifier of pre-existing intramural stress.

Perivascular and juxtavascular microglia are geographically separate subsets that have direct physical interaction with the vascular basement membrane and pericyte processes. In response to vascular Aβ, these cells engage TLR4 signaling [140] and metabolic remodeling driven by the mTOR–HIF-1α axis, moving from oxidative phosphorylation to aerobic glycolysis and developing a pro-inflammatory phagocytic phenotype [141]. The conventional M1/M2 dichotomy, while increasingly considered as an oversimplification [142,143], depicts a functional asymmetry with evident ramifications in this perivascular zone. Early TREM2-dependent responses promote microglial clustering at the vessel wall and immersion of vascular Aβ, partially maintaining mural stability. However, deletion of TREM2 significantly reduces this perivascular response and exacerbates CAA-associated vascular disease [144]. Chronic activation of the NLRP3 inflammasome causes IL-1β release, impaired microglial Aβ clearance, and increased neuroinflammation, leading to vascular and mural cell impairment [145]. Because perivascular microglia signal across a distance of only a few micrometers from the contractile mural compartment, their secretome operates on SMC and pericytes at near-paracrine concentrations, providing a mechanistic basis for the disproportionate vulnerability of the vessel wall observed in CAA and AD.

Astrocytes contribute to this same intramural axis through an anatomically complementary route. Astrocytic endfeet cover nearly the entire cerebrovascular tree and form the outermost layer of the gliovascular unit, in intimate contact with the parenchymal basement membrane that envelops SMCs and pericytes. Reactive transformation of astrocytes in AD—marked by glial fibrillary acidic protein (GFAP) upregulation, process hypertrophy, STAT3 activation, and altered fatty acid handling with mitochondrial acetyl-CoA accumulation [143,146]—therefore translates directly into altered signaling at the endfoot–mural cell interface. Reactive astrocytes secrete cytokines, lose polarized AQP4 expression at the perivascular endfoot membrane, and remodel the extracellular matrix through MMP/TIMP release; each of these changes acts on a structure (the basement membrane, the perivascular drainage pathway, the gliovascular coupling apparatus) on which mural cell function depends. Loss of perivascular AQP4 polarization, in particular, impairs glymphatic solute exchange, slows interstitial Aβ clearance, and is associated with accelerated vascular and parenchymal Aβ deposition in both rodent models and human AD cortex [129,147,148], providing a direct astrocyte-to-mural-cell mechanistic link. On this view, astrocytic reactivity is not a parenchymal event running in parallel with intramural pathology—it is itself a modulator of mural cell contractility, of barrier-support signaling, and of the basement membrane scaffold in which contractile cells are embedded.

Microglia–astrocyte interaction within the perivascular niche further amplifies the intramural damage. Activated microglia release TNF, IL-1α, and C1q, which are together necessary and sufficient to convert neighboring astrocytes to a neurotoxic A1-like state that is abundant in AD cortex [149]. This conversion is bidirectional and operates in part through extracellular vesicle (EV) trafficking: astrocyte-derived EVs carrying miR-873a-5p suppress microglial NF-κB and ERK signaling and promote an anti-inflammatory phenotype [150], whereas astrocyte EVs carrying miR-138 activate microglia via endosomal TLR7 [151]. EVs released by astrocytes from AD model mice and from sporadic and familial AD patients directly impair both neuroglial and vascular components of the NVU [152], establishing that this crosstalk is operative in AD itself. Because it takes place in the same perivascular microenvironment that houses pericytes and SMCs, the cytokines, miRNAs, and lncRNAs exchanged between glia simultaneously bathe the contractile mural compartment. The self-amplifying glial loop that drives synaptic failure in the parenchyma therefore also drives contractile dysfunction and basement membrane remodeling within the vessel wall.

**Figure 6:**
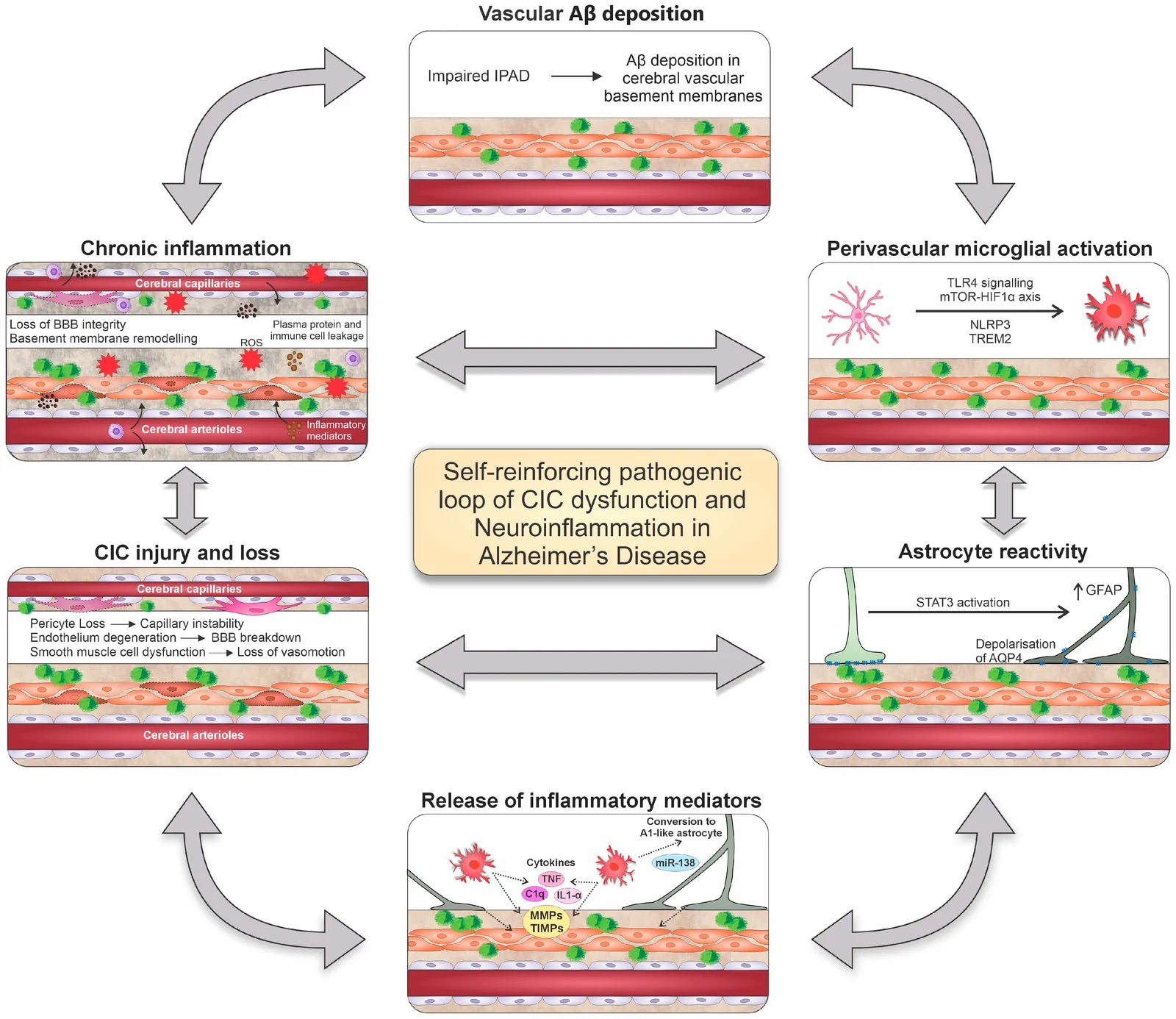
Proposed model illustrating the bidirectional interactions between CIC dysfunction and neuroinflammation during AD progression. Impaired IPAD promotes vascular Aβ accumulation within cerebral vascular basement membranes, triggering perivascular microglial activation and reactive astrocytosis. Activated glial cells release pro-inflammatory cytokines, MMPs, reactive oxygen species (ROS), and other inflammatory mediators that exacerbate blood–brain barrier disruption, basement membrane remodeling, and mural cell injury. Progressive degeneration of pericytes, endothelial cells, and vascular SMCs further impairs vascular function and Aβ clearance, establishing a self-perpetuating cycle of neurovascular dysfunction, chronic inflammation, cerebral amyloid angiopathy, and neurodegeneration. Figure created with CorelDRAW Graphics Suite 2025 (Alludo, Ottawa, ON, Canada).

Neuroinflammation can therefore be placed both upstream and downstream of CIC dysfunction. Early CIC degeneration permits BBB leakage and admits plasma proteins, fibrinogen, and complement into the perivascular compartment, where they activate perivascular microglia and recruit reactive astrocytes. Chronic hypoperfusion and oxidative stress—themselves consequences of impaired CIC vasomotion—promote protease activation and matrix remodeling within the vessel wall, further compromising the contractile and barrier-support programs of the surviving mural cells. In late-onset disease, this coupling permits glial activation to transition from a protective response to a self-sustaining amplifier of mural injury, perfusion deficit, and clearance failure. The bidirectional interactions between CIC dysfunction and neuroinflammation during AD progression are summarized in Figure 6.

## 12. Therapeutic Targeting of Intramural Cells: Current Strategies and Future Directions

Since IPAD is driven by slow vasomotion of arteriolar SMCs [87,128], pharmacological preservation of contractile function is the most direct therapy. Mechanism-directed candidates include voltage-dependent calcium signaling modulators—voltage-dependent calcium channel involvement declines along the arteriole–capillary continuum, with full dependence in SMCs but partial or absent dependence in transition-zone and distal pericytes [79]; and agents acting on the NO-cGMP pathway to sustain vasomotion and conducted vasodilation.

A second axis targets pericyte–endothelial signaling and BBB maintenance. The PDGF-B-PDGFRβ signaling axis sustains capillary pericytes, and disrupting it leads to BBB breakdown and neurovascular dysfunction [153,154]. This axis is downregulated in human AD cortex, providing a translational rationale for strategies that stabilize PDGFRβ signaling or reduce its proteasomal turnover [155]. The CypA–MMP-9 pathway in pericytes is the primary effector of APOE4-driven BBB breakdown: APOE4 activates a proinflammatory CypA–NF-κB–MMP-9 cascade that degrades tight junctions and accelerates pericyte loss [20], and this pathway is upregulated in AD APOE4 > APOE3 carriers in both post-mortem tissue [21] and CSF, where its activity correlates with the pericyte injury marker soluble PDGFRβ and predicts cognitive decline independently of amyloid and tau [22].

The third axis is to restore intramural clearance. According to Hartz et al. [132] and Storck et al. [133], a tractable upstream mechanism is provided by ubiquitin-mediated degradation of P-gp under Aβ and inflammatory stress. P-glycoprotein-mediated Aβ efflux decreases in normal aging and is further reduced in early AD [156,157]. Approaches under investigation include pregnane X receptor agonism (rifampicin, with hepatic-toxicity cautions), xanthine-class P-gp modulators, and emerging UPS-stabilizing strategies that limit transporter turnover under oxidative stress. LRP-1-mediated peripheral Aβ sequestration provides a complementary route [139]. These clearance-directed therapies work on the CIC system’s endothelium and pericyte compartments; thus, they complement rather than replace vasomotion-preserving methods.

Second-generation anti-amyloid monoclonal antibodies are emerging as viable therapeutic options for patients with mild AD. While passive immunotherapy lowers the plaque Aβ, there is still a high risk of amyloid-related imaging abnormality (ARIA) in approximately 25% of treated patients [158,159]. Post-mortem human studies have correlated ARIA with worsening of CAA in treated individuals, especially those possessing at least an allele of APOE4 [160,161].

The breakdown of parenchymal Aβ plaques and their subsequent mobilization along IPAD pathways may further increase the Aβ vascular burden and exacerbate CAA-related pathology in some individuals, while also further worsening the integrity of the CIC. Therapeutic approaches of CIC such as carbonic anhydrase inhibitors may restore mitochondrial function in the aging CICs [162,163].

Current guidelines stratify ARIA risk by APOE genotype and baseline microbleed burden [57]. Within the CIC framework, this stratification is most naturally interpreted as a CIC-vulnerability stratification. CSF sPDGFRβ and dynamic contrast-enhanced MRI of hippocampal BBB permeability [32], together with verapamil-PET indices of BBB P-gp activity, offer practical biomarkers for identifying patients with high baseline CIC injury who may benefit most from CIC-targeted therapy or face higher ARIA risk under aggressive anti-amyloid regimens. Lifestyle interventions (aerobic exercise, Mediterranean and DASH dietary patterns, midlife blood–pressure control) act on multiple components of the CIC compartment simultaneously and remain the main interventions for which large-scale evidence of dementia risk reduction exists.

A clear translational gap separates the preclinical strategies directed at PDGFRβ signaling and P-glycoprotein-mediated clearance from clinical application; neither has entered human testing. Cilostazol, a phosphodiesterase III inhibitor, restores vascular reactivity and hemodynamic reserve, promotes IPAD of Aβ, reduces the degenerative changes of CIC and prevents the decline of cognitive performance in the Tg-SwDI mouse model of CAA [164].

Cilostazol and isosorbide mononitrate are well-tolerated individually and together and in addition to conventional secondary stroke prevention, appear to improve CIC function, cognitive and neuroimaging secondary outcomes [165]. All the available evidence from the LACI-1 trial supports wider testing of cilostazol and isosorbide mononitrate to prevent worsening of cerebral small vessel disease, a common cause of stroke, cognitive decline and dementia. This combination within LACI-2 promises to restore also the function of dysregulated CIC [166,167].

## 13. Testing the Framework: Predictions, Criteria and Biomarkers

As the CIC concept is advanced as a working hypothesis, we state what would count against it. Table 1 gives five predictions, with the design that would support each, the observation that would refute it, and an existing human biomarker through which it could be tested prospectively. P1 and P3 are decisive, the first for temporal ordering and the second for explanatory independence beyond amyloid and tau. Vasomotion, the variable at the center of the framework, still lacks a validated human measurement standard, and establishing one is a prerequisite for testing P1 directly.

**Table 1:** Predictions of the CIC framework, with supporting designs, refuting observations and candidate biomarkers.

Prediction	Supported by	Refuted by	Candidate Biomarker
P1. Loss of vasomotion and IPAD precedes microbleeds, arteriolar α-SMA loss and impaired cerebrovascular reactivity (CVR)	VSMC contraction drives IPAD [40,87,115]; in APP23 mice vasomotion declines before microbleeds, α-SMA loss and reduced CVR [86].	Vasomotion preserved once these markers are established, or shown to decline only after them.	Low-frequency hemodynamic oscillation imaging with CVR (investigational; no validated clinical standard) [86,113].
P2. IPAD impairment is mechanistically distinct from glymphatic dysfunction and endothelial transcytosis failure	IPAD depends on arteriolar contractility along the basement-membrane route [87,115,128]; AQP4 loss impairs glymphatic influx without directly targeting IPAD [43,129,147,148].	Selective loss of vasomotion leaves IPAD intact, or the three routes prove experimentally inseparable.	Enlarged perivascular spaces [131]; CSF/ISF tracer imaging (research use) [43,131].
P3. CIC injury predicts cognitive decline independently of amyloid and tau	CSF sPDGFRβ and hippocampal K-trans mark BBB breakdown in early cognitive dysfunction irrespective of Aβ/tau status [32]; BBB dysfunction predicts decline in APOEε4 carriers [22]; permeability rises with age [93].	No incremental prediction beyond amyloid, tau and vascular risk factors in adequately powered cohorts.	CSF sPDGFRβ [22,32]; DCE-MRI hippocampal K-trans [32,93].
P4. APOEε4-related injury follows a spatial gradient, greatest in capillary pericytes.	APOEε4 activates CypA–MMP-9 and disrupts the BBB [20]; pericyte degeneration is accelerated in ε4 carriers in proportion to BBB breakdown [21]. Segment-level comparison remains untested.	Injury equivalent across capillaries, arterioles and larger vessels, or VSMCs affected before pericytes.	CSF CypA and MMP-9 [22]; vascular proteomic panels.
P5. Preserving intramural function improves outcome by a vascular rather than a direct anti-amyloid mechanism.	Cilostazol restores reactivity, promotes IPAD and preserves cognition in Tg-SwDI mice [164]; cilostazol with isosorbide mononitrate improves hemodynamics (LACI-1) [165] and clinical outcomes (LACI-2) [166,167].	Benefit fully explained by change in amyloid or tau burden, or absent despite confirmed vascular target engagement.	CVR and hemodynamic measures [165]; perivascular spaces and cSVD markers [131]; ^11^C-verapamil PET where clearance-directed agents are tested [156,157].

## 14. Limitations, Methodological Constraints and Translational Gaps

The CIC framework is advanced as a working hypothesis, and its evidential foundation carries constraints that should be stated explicitly. These fall into three categories: the technical limits of the methods available to study intramural cells, the species gap between the rodent models that supply most mechanistic data and human disease, and the incomplete characterization of age and sex as modifiers of intramural vulnerability.

### 14.1. Methodological Limitations in the Study of Cerebral Intramural Cells

Separating pericyte from SMC function in situ remains difficult: α-SMA expression varies as a continuum along the arteriole–capillary axis rather than as a categorical marker, and a proportion is lost during conventional fixation, so contractile capacity may be underestimated [168]. Driver lines based on PDGFRβ, NG2 and Myh11 each label additional populations, and inconsistent subtype nomenclature is a recognized source of conflicting results [45,49]. Distinguishing genuine pathology from imaging artifact is a further constraint: hippocampal DCE-MRI K-trans lies close to the noise floor and depends on the pharmacokinetic model chosen [32], perivascular space quantification varies with field strength and sequence [131], and low-frequency hemodynamic oscillations proposed as vasomotion readouts are confounded by systemic blood pressure and respiratory variation. No protocol for these measures is standardized across sites, and CSF sPDGFRβ lacks certified reference material [22].

### 14.2. Species Differences

Murine Aβ differs from the human peptide at three residues and does not spontaneously aggregate, so all amyloid models require transgenic overexpression; most develop neither tangles nor substantial neuronal loss, and the rodent lifespan compresses decades of human vascular aging into two to three years. IPAD path lengths and arterial pulsatility differ correspondingly, so drainage efficiency measured in mice cannot be assumed to scale to the human brain [87]. Commonly used CAA models such as Tg-SwDI are capillary-predominant, whereas most sporadic human CAA is arteriolar-predominant [164]. Most directly, single-nucleus profiling of the human brain vasculature has shown that many mural cell zonation and subtype markers differ between species, and that a substantial proportion of AD risk genes are expressed in human vascular cells but predominantly in murine microglia [169]; rodent-derived subtype assignments should therefore be treated as provisional [36,84]. Conversely, the dependence of periarterial drainage on arteriolar contractile activity, the link between pericyte injury and BBB breakdown, and the APOEε4–CypA–MMP-9 mechanism are each supported by human as well as animal data [20,21,22,32], so the core proposition is likely to translate even where the subtype-level architecture is not.

### 14.3. Age and Sex

Age-related decline in pericyte coverage [61,62], basement membrane thickening [4], capillary loss and string vessel formation [109], and arterial stiffening [50] all reduce the driving force for IPAD [83], and these changes are not uniform along the vascular tree; venular mural cells in particular remain almost unstudied in aging. Sex effects are less characterized still. Women account for approximately two-thirds of prevalent AD cases [170]; the APOEε4 association with AD is stronger in women than in men between roughly 65 and 75 years [171], and women show greater entorhinal tau at comparable amyloid burden [172]. Whether any of this is mediated through the intramural compartment is unknown: sex-stratified data on pericyte density, sPDGFRβ trajectories, vasomotion and IPAD efficiency are largely absent, and much of the rodent work reviewed here used male animals only or did not report by sex.

## 15. Conclusions

The CIC framework supports a unified vascular–parenchymal disease logic in which aging-related deterioration of the vessel wall acts as a permissive substrate for Aβ and tau to become clinically consequential. Intramural dysfunction can begin with subtle impairments in vasomotion and conducted dilation, producing microhemodynamic inefficiency and regional vulnerability in low-reserve territories such as white matter and hippocampus. CIC-driven deterioration of BBB signaling and basement membrane homeostasis can promote early barrier leakage and impede intramural and perivascular clearance, favoring vascular Aβ retention and progressive accumulation of toxic metabolites. As amyloid increases, direct effects on pericyte tone and capillary stability further amplify perfusion and clearance deficits. These vascular-wall alterations then converge with glial activation programs to drive synaptic loss, network disconnection, and neurodegeneration, providing a mechanistic explanation for why similar plaque burdens can yield divergent clinical outcomes. While there is clear evidence on the effects of sex and age on the incidence of dementia, most studies concentrate on endothelial cells rather than CIC. There is a clear need for such studies in the near future. Apart from transcriptomic studies, there is still a lack of quantitative data on the pathophysiology of CIC in particular in relation to translational studies. Observed through a CIC-centered lens, vascular aging is no longer a comorbidity of AD but a cellularly grounded driver of disease expression and progression.

## Data Availability

Any data relevant to this review can be obtained from the corresponding author upon reasonable request.

## Abbreviations

The following abbreviations are used in this manuscript:

Aβ	amyloid-β
AD	Alzheimer’s disease
CAA	cerebral amyloid angiopathy
IPAD	intramural periarterial drainage
CICs	cerebral intramural cells
BBB	blood–brain barrier
SMC	smooth muscle cell
CBF	cerebral blood flow
APP	amyloid precursor protein
BACE1	beta-site amyloid precursor protein cleaving enzyme 1
SCAA	sporadic cerebral amyloid angiopathy
APOE	apolipoprotein E
APOɛ4	apolipoprotein ɛ4
NVU	neurovascular unit
CSF	cerebrospinal fluid
ADNI	Alzheimer’s Disease Neuroimaging Initiative
NVC	neurovascular coupling
ACT	arteriole–capillary transition
VCID	vascular contributions to cognitive impairment and dementia
VaD	vascular dementia
PSD	post-stroke dementia
COL4	collagen 4
cSVD	cerebral small vessel disease
CADASIL	cerebral autosomal-dominant arteriopathy with subcortical infarcts and leukoencepha-lopathy
GOM	granular osmiophilic material
Atp13a5	ATPase 13A5
CNS	central nervous system
BECs	brain endothelial cells
ROS	reactive oxygen species
ACH	amyloid cascade hypothesis
GFAP	glial fibrillary acidic protein
ARIA	amyloid-related imaging abnormalities
CVR	cerebrovascular reactivity
P-gp	P-glycoprotein
α-SMC	α-smooth muscle actin

## References

[1] Iadecola C. (2004). Neurovascular Regulation in the Normal Brain and in Alzheimer’s Disease. Nat. Rev. Neurosci..

[2] Selkoe D.J., Schenk D. (2003). Alzheimer’s Disease: Molecular Understanding Predicts Amyloid-Based Therapeutics. Annu. Rev. Pharmacol. Toxicol..

[3] De Strooper B., Iwatsubo T., Wolfe M.S. (2012). Presenilins and γ-Secretase: Structure, Function, and Role in Alzheimer Disease. Cold Spring Harb. Perspect. Med..

[4] Farkas E., Luiten P.G. (2001). Cerebral Microvascular Pathology in Aging and Alzheimer’s Disease. Prog. Neurobiol..

[5] Vemuri P., Lesnick T.G., Przybelski S.A., Knopman D.S., Preboske G.M., Kantarci K., Raman M.R., Machulda M.M., Mielke M.M., Lowe V.J. (2015). Vascular and Amyloid Pathologies Are Independent Predictors of Cognitive Decline in Normal Elderly. Brain.

[6] Iturria-Medina Y., Sotero R.C., Toussaint P.J., Mateos-Pérez J.M., Evans A.C. (2016). Early Role of Vascular Dysregulation on Late-Onset Alzheimer’s Disease Based on Multifactorial Data-Driven Analysis. Nat. Commun..

[7] Zheng H., Koo E.H. (2011). Biology and Pathophysiology of the Amyloid Precursor Protein. Mol. Neurodegener..

[8] Lemere C.A., Blusztajn J.K., Yamaguchi H., Wisniewski T., Saido T.C., Selkoe D.J. (1996). Sequence of Deposition of Heterogeneous Amyloid Beta-Peptides and APO E in Down Syndrome: Implications for Initial Events in Amyloid Plaque Formation. Neurobiol. Dis..

[9] Trejo-Lopez J.A., Yachnis A.T., Prokop S. (2022). Neuropathology of Alzheimer’s Disease. Neurotherapeutics.

[10] Tanzi R.E. (2012). The Genetics of Alzheimer Disease. Cold Spring Harb. Perspect. Med..

[11] Freitag K., Sterczyk N., Wendlinger S., Obermayer B., Schulz J., Farztdinov V., Mülleder M., Ralser M., Houtman J., Fleck L. (2022). Spermidine Reduces Neuroinflammation and Soluble Amyloid Beta in an Alzheimer’s Disease Mouse Model. J. Neuroinflammation.

[12] Dickson D.W., Crystal H.A., Mattiace L.A., Masur D.M., Blau A.D., Davies P., Yen S.H., Aronson M.K. (1992). Identification of Normal and Pathological Aging in Prospectively Studied Nondemented Elderly Humans. Neurobiol. Aging.

[13] Case N.F., Charlton A., Zwiers A., Batool S., McCreary C.R., Hogan D.B., Ismail Z., Zerna C., Coutts S.B., Frayne R. (2016). Cerebral Amyloid Angiopathy Is Associated with Executive Dysfunction and Mild Cognitive Impairment. Stroke.

[14] Tsering W., Prokop S. (2024). Neuritic Plaques—Gateways to Understanding Alzheimer’s Disease. Mol. Neurobiol..

[15] Iqbal K., Flory M., Khatoon S., Soininen H., Pirttila T., Lehtovirta M., Alafuzoff I., Blennow K., Andreasen N., Vanmechelen E. (2005). Subgroups of Alzheimer’s Disease Based on Cerebrospinal Fluid Molecular Markers. Ann. Neurol..

[16] Iqbal K., Liu F., Gong C.-X. (2014). Alzheimer Disease Therapeutics: Focus on the Disease and Not Just Plaques and Tangles. Biochem. Pharmacol..

[17] Knopman D.S., Amieva H., Petersen R.C., Chételat G., Holtzman D.M., Hyman B.T., Nixon R.A., Jones D.T. (2021). Alzheimer Disease. Nat. Rev. Dis. Prim..

[18] Roses A.D. (2006). On the Discovery of the Genetic Association of Apolipoprotein E Genotypes and Common Late-Onset Alzheimer Disease. J. Alzheimer’s Dis..

[19] Jackson R.J., Hyman B.T., Serrano-Pozo A. (2024). Multifaceted Roles of APOE in Alzheimer Disease. Nat. Rev. Neurol..

[20] Bell R.D., Winkler E.A., Singh I., Sagare A.P., Deane R., Wu Z., Holtzman D.M., Betsholtz C., Armulik A., Sallstrom J. (2012). Apolipoprotein E Controls Cerebrovascular Integrity via Cyclophilin A. Nature.

[21] Halliday M.R., Rege S.V., Ma Q., Zhao Z., Miller C.A., Winkler E.A., Zlokovic B.V. (2016). Accelerated Pericyte Degeneration and Blood-Brain Barrier Breakdown in Apolipoprotein E4 Carriers with Alzheimer’s Disease. J. Cereb. Blood Flow. Metab..

[22] Montagne A., Nation D.A., Sagare A.P., Barisano G., Sweeney M.D., Chakhoyan A., Pachicano M., Joe E., Nelson A.R., D’Orazio L.M. (2020). APOE4 Leads to Blood–Brain Barrier Dysfunction Predicting Cognitive Decline. Nature.

[23] Tomlinson B.E., Blessed G., Roth M. (1970). Observations on the Brains of Demented Old People. J. Neurol. Sci..

[24] Mormino E.C., Betensky R.A., Hedden T., Schultz A.P., Amariglio R.E., Rentz D.M., Johnson K.A., Sperling R.A. (2014). Synergistic Effect of β-Amyloid and Neurodegeneration on Cognitive Decline in Clinically Normal Individuals. JAMA Neurol..

[25] Jansen W.J., Ossenkoppele R., Knol D.L., Tijms B.M., Scheltens P., Verhey F.R.J., Visser P.J., Aalten P., Aarsland D., Amyloid Biomarker Study Group (2015). Prevalence of Cerebral Amyloid Pathology in Persons without Dementia: A Meta-Analysis. JAMA.

[26] Ossenkoppele R., Pichet Binette A., Groot C., Smith R., Strandberg O., Palmqvist S., Stomrud E., Tideman P., Ohlsson T., Jögi J. (2022). Amyloid and Tau PET-Positive Cognitively Unimpaired Individuals Are at High Risk for Future Cognitive Decline. Nat. Med..

[27] Schneider J.A., Arvanitakis Z., Bang W., Bennett D.A. (2007). Mixed Brain Pathologies Account for Most Dementia Cases in Community-Dwelling Older Persons. Neurology.

[28] Arvanitakis Z., Leurgans S.E., Wang Z., Wilson R.S., Bennett D.A., Schneider J.A. (2011). Cerebral Amyloid Angiopathy Pathology and Cognitive Domains in Older Persons. Ann. Neurol..

[29] Boyle P.A., Yu L., Nag S., Leurgans S., Wilson R.S., Bennett D.A., Schneider J.A. (2015). Cerebral Amyloid Angiopathy and Cognitive Outcomes in Community-Based Older Persons. Neurology.

[30] Godrich D., Pasteris J., Martin E.R., Rundek T., Schellenberg G., Foroud T., Vance J.M., Pericak-Vance M.A., Cuccaro M.L., Scott W.K. (2024). Cerebral Amyloid Angiopathy Impacts Neurofibrillary Tangle Burden and Cognition. Brain Commun..

[31] Natté R., Maat-Schieman M.L.C., Haan J., Bornebroek M., Roos R.A.C., Van Duinen S.G. (2001). Dementia in Hereditary Cerebral Hemorrhage with Amyloidosis-Dutch Type Is Associated with Cerebral Amyloid Angiopathy but Is Independent of Plaques and Neurofibrillary Tangles. Ann. Neurol..

[32] Nation D.A., Sweeney M.D., Montagne A., Sagare A.P., D’Orazio L.M., Pachicano M., Sepehrband F., Nelson A.R., Buennagel D.P., Harrington M.G. (2019). Blood–Brain Barrier Breakdown Is an Early Biomarker of Human Cognitive Dysfunction. Nat. Med..

[33] Wang J., Fan D.-Y., Li H.-Y., He C.-Y., Shen Y.-Y., Zeng G.-H., Chen D.-W., Yi X., Ma Y.-H., Yu J.-T. (2022). Dynamic Changes of CSF sPDGFRβ during Ageing and AD Progression and Associations with CSF ATN Biomarkers. Mol. Neurodegener..

[34] Hristovska I., Pichet Binette A., Kumar A., Wennström M., Gaiteri C., Karlsson L., Strandberg O., Janelidze S., van Westen D., Stomrud E. (2026). Identification of Distinct and Shared Biomarker Panels in Different Manifestations of Cerebral Small-Vessel Disease through Proteomic Profiling. Nat. Aging.

[35] Xiong C., Tang Y., Chen J., Fan M., Wei L., Dong Z., Lai X., Men X., Chen Q., Li D. (2025). Transplantation of hiPSC-Derived Pericytes Rescues Alzheimer’s Disease Phenotypes in APOE4/4 Mice through IGF2-Rich Apoptotic Vesicles. Transl. Neurodegener..

[36] Vanlandewijck M., He L., Mäe M.A., Andrae J., Ando K., Del Gaudio F., Nahar K., Lebouvier T., Laviña B., Gouveia L. (2018). A Molecular Atlas of Cell Types and Zonation in the Brain Vasculature. Nature.

[37] Iadecola C. (2017). The Neurovascular Unit Coming of Age: A Journey through Neurovascular Coupling in Health and Disease. Neuron.

[38] Alkayed N.J., Cipolla M.J. (2023). The Ever-Evolving Concept of the Neurovascular Unit. Stroke.

[39] Gong Y., Wu M., Huang Y., He X., Yuan J., Dang B. (2025). Research Developments in the Neurovascular Unit and the Blood-brain Barrier (Review). Biomed. Rep..

[40] Carare R.O., Aldea R., Bulters D., Alzetani A., Birch A.A., Richardson G., Weller R.O. (2020). Vasomotion Drives Periarterial Drainage of Aβ from the Brain. Neuron.

[41] Sun Y., Liu E., Pei Y., Yao Q., Ma H., Mu Y., Wang Y., Zhang Y., Yang X., Wang X. (2022). The Impairment of Intramural Periarterial Drainage in Brain after Subarachnoid Hemorrhage. Acta Neuropathol. Commun..

[42] Ding Z., Fan X., Zhang Y., Yao M., Wang G., Dong Y., Liu J., Song W. (2023). The Glymphatic System: A New Perspective on Brain Diseases. Front. Aging Neurosci..

[43] Gomolka R.S., Hablitz L.M., Mestre H., Giannetto M., Du T., Hauglund N.L., Xie L., Peng W., Martinez P.M., Nedergaard M. (2023). Loss of Aquaporin-4 Results in Glymphatic System Dysfunction via Brain-Wide Interstitial Fluid Stagnation. eLife.

[44] Bonnar O., Eyre B., van Veluw S.J. (2025). Perivascular Brain Clearance as a Therapeutic Target in Cerebral Amyloid Angiopathy and Alzheimer’s Disease. Neurotherapeutics.

[45] Hartmann D.A., Coelho-Santos V., Shih A.Y. (2022). Pericyte Control of Blood Flow Across Microvascular Zones in the Central Nervous System. Annu. Rev. Physiol..

[46] Ojo J.O., Reed J.M., Crynen G., Vallabhaneni P., Evans J., Shackleton B., Eisenbaum M., Ringland C., Edsell A., Mullan M. (2021). Molecular Pathobiology of the Cerebrovasculature in Aging and in Alzheimers Disease Cases with Cerebral Amyloid Angiopathy. Front. Aging Neurosci..

[47] Fisher R.A., Miners J.S., Love S. (2022). Pathological Changes within the Cerebral Vasculature in Alzheimer’s Disease: New Perspectives. Brain Pathol..

[48] Chen S., Guo D., Zhu Y., Xiao S., Xie J., Zhang Z., Hu Y., Huang J., Ma X., Ning Z. (2024). Amyloid β Oligomer Induces Cerebral Vasculopathy via Pericyte-Mediated Endothelial Dysfunction. Alzheimers Res. Ther..

[49] Hartmann D.A., Berthiaume A.-A., Grant R.I., Harrill S.A., Koski T., Tieu T., McDowell K.P., Faino A.V., Kelly A.L., Shih A.Y. (2021). Brain Capillary Pericytes Exert a Substantial but Slow Influence on Blood Flow. Nat. Neurosci..

[50] Hayes G., Pinto J., Sparks S.N., Wang C., Suri S., Bulte D.P. (2022). Vascular Smooth Muscle Cell Dysfunction in Neurodegeneration. Front. Neurosci..

[51] Aguilar-Pineda J.A., Vera-Lopez K.J., Shrivastava P., Chávez-Fumagalli M.A., Nieto-Montesinos R., Alvarez-Fernandez K.L., Goyzueta Mamani L.D., Davila Del-Carpio G., Gomez-Valdez B., Miller C.L. (2021). Vascular Smooth Muscle Cell Dysfunction Contribute to Neuroinflammation and Tau Hyperphosphorylation in Alzheimer Disease. iScience.

[52] Profaci C.P., Munji R.N., Pulido R.S., Daneman R. (2020). The Blood-Brain Barrier in Health and Disease: Important Unanswered Questions. J. Exp. Med..

[53] You T.-Y., Dong Q., Cui M. (2023). Emerging Links between Cerebral Blood Flow Regulation and Cognitive Decline: A Role for Brain Microvascular Pericytes. Aging Dis..

[54] Inoue Y., Shue F., Bu G., Kanekiyo T. (2023). Pathophysiology and Probable Etiology of Cerebral Small Vessel Disease in Vascular Dementia and Alzheimer’s Disease. Mol. Neurodegener..

[55] Iadecola C., Gottesman R.F. (2018). Cerebrovascular Alterations in Alzheimer’s Disease: Incidental or Pathogenic?. Circ. Res..

[56] Brenowitz W.D., Nelson P.T., Besser L.M., Heller K.B., Kukull W.A. (2015). Cerebral Amyloid Angiopathy and Its Co-Occurrence with Alzheimer’s Disease and Other Cerebrovascular Neuropathologic Changes. Neurobiol. Aging.

[57] Greenberg S.M., Bacskai B.J., Hernandez-Guillamon M., Pruzin J., Sperling R., van Veluw S.J. (2020). Cerebral Amyloid Angiopathy and Alzheimer Disease—One Peptide, Two Pathways. Nat. Rev. Neurol..

[58] Jäkel L., De Kort A.M., Klijn C.J.M., Schreuder F.H.B.M., Verbeek M.M. (2022). Prevalence of Cerebral Amyloid Angiopathy: A Systematic Review and Meta-Analysis. Alzheimers Dement..

[59] Hannawi Y. (2024). Cerebral Small Vessel Disease: A Review of the Pathophysiological Mechanisms. Transl. Stroke Res..

[60] Markus H.S., Joutel A. (2025). The Pathogenesis of Cerebral Small Vessel Disease and Vascular Cognitive Impairment. Physiol. Rev..

[61] Ding R., Hase Y., Ameen-Ali K.E., Ndung’u M., Stevenson W., Barsby J., Gourlay R., Akinyemi T., Akinyemi R., Uemura M.T. (2020). Loss of Capillary Pericytes and the Blood-Brain Barrier in White Matter in Poststroke and Vascular Dementias and Alzheimer’s Disease. Brain Pathol..

[62] Ding R., Hase Y., Burke M., Foster V., Stevenson W., Polvikoski T., Kalaria R.N. (2021). Loss with Ageing but Preservation of Frontal Cortical Capillary Pericytes in Post-Stroke Dementia, Vascular Dementia and Alzheimer’s Disease. Acta Neuropathol. Commun..

[63] Attems J., Lintner F., Jellinger K.A. (2004). Amyloid β Peptide 1-42 Highly Correlates with Capillary Cerebral Amyloid Angiopathy and Alzheimer Disease Pathology. Acta Neuropathol..

[64] Thal D.R., Ghebremedhin E., Rüb U., Yamaguchi H., Del Tredici K., Braak H. (2002). Two Types of Sporadic Cerebral Amyloid Angiopathy. J. Neuropathol. Exp. Neurol..

[65] Thal D.R., Griffin W.S.T., de Vos R.A.I., Ghebremedhin E. (2008). Cerebral Amyloid Angiopathy and Its Relationship to Alzheimer’s Disease. Acta Neuropathol..

[66] Mandybur T.I. (1986). Cerebral Amyloid Angiopathy: The Vascular Pathology and Complications. J. Neuropathol. Exp. Neurol..

[67] Vonsattel J.P., Myers R.H., Hedley-Whyte E.T., Ropper A.H., Bird E.D., Richardson E.P. (1991). Cerebral Amyloid Angiopathy without and with Cerebral Hemorrhages: A Comparative Histological Study. Ann. Neurol..

[68] Thal D.R., Capetillo-Zarate E., Larionov S., Staufenbiel M., Zurbruegg S., Beckmann N. (2009). Capillary Cerebral Amyloid Angiopathy Is Associated with Vessel Occlusion and Cerebral Blood Flow Disturbances. Neurobiol. Aging.

[69] Charidimou A., Boulouis G., Frosch M.P., Baron J.-C., Pasi M., Albucher J.F., Banerjee G., Barbato C., Bonneville F., Brandner S. (2022). The Boston Criteria Version 2.0 for Cerebral Amyloid Angiopathy: A Multicentre, Retrospective, MRI-Neuropathology Diagnostic Accuracy Study. Lancet Neurol..

[70] Duering M., Biessels G.J., Brodtmann A., Chen C., Cordonnier C., de Leeuw F.-E., Debette S., Frayne R., Jouvent E., Rost N.S. (2023). Neuroimaging Standards for Research into Small Vessel Disease-Advances since 2013. Lancet Neurol..

[71] Yang Y., Qian Y., Huang J., Luo Y. (2026). Cerebral Small Vessel Disease and Blood-Brain Barrier Disruption: Mechanisms and Therapeutic Strategies in Cognitive Impairment. Transl. Stroke Res..

[72] Blinder P., Shih A.Y., Rafie C., Kleinfeld D. (2010). Topological Basis for the Robust Distribution of Blood to Rodent Neocortex. Proc. Natl. Acad. Sci. USA.

[73] Nguyen J., Nishimura N., Fetcho R.N., Iadecola C., Schaffer C.B. (2011). Occlusion of Cortical Ascending Venules Causes Blood Flow Decreases, Reversals in Flow Direction, and Vessel Dilation in Upstream Capillaries. J. Cereb. Blood Flow. Metab..

[74] Taylor Z.J., Hui E.S., Watson A.N., Nie X., Deardorff R.L., Jensen J.H., Helpern J.A., Shih A.Y. (2016). Microvascular Basis for Growth of Small Infarcts Following Occlusion of Single Penetrating Arterioles in Mouse Cortex. J. Cereb. Blood Flow. Metab..

[75] Grant R.I., Hartmann D.A., Underly R.G., Berthiaume A.-A., Bhat N.R., Shih A.Y. (2019). Organizational Hierarchy and Structural Diversity of Microvascular Pericytes in Adult Mouse Cortex. J. Cereb. Blood Flow. Metab..

[76] Kucharz K., Kristensen K., Johnsen K.B., Lund M.A., Lønstrup M., Moos T., Andresen T.L., Lauritzen M.J. (2021). Post-Capillary Venules Are the Key Locus for Transcytosis-Mediated Brain Delivery of Therapeutic Nanoparticles. Nat. Commun..

[77] Berthiaume A.-A., Grant R.I., McDowell K.P., Underly R.G., Hartmann D.A., Levy M., Bhat N.R., Shih A.Y. (2018). Dynamic Remodeling of Pericytes In Vivo Maintains Capillary Coverage in the Adult Mouse Brain. Cell Rep..

[78] Ornelas S., Berthiaume A.-A., Bonney S.K., Coelho-Santos V., Underly R.G., Kremer A., Guérin C.J., Lippens S., Shih A.Y. (2021). Three-Dimensional Ultrastructure of the Brain Pericyte-Endothelial Interface. J. Cereb. Blood Flow. Metab..

[79] Klug N.R., Sancho M., Gonzales A.L., Heppner T.J., O’Brien R.I.C., Hill-Eubanks D., Nelson M.T. (2023). Intraluminal Pressure Elevates Intracellular Calcium and Contracts CNS Pericytes: Role of Voltage-Dependent Calcium Channels. Proc. Natl. Acad. Sci. USA.

[80] Glück C., Ferrari K.D., Binini N., Keller A., Saab A.S., Stobart J.L., Weber B. (2021). Distinct Signatures of Calcium Activity in Brain Mural Cells. eLife.

[81] Erdener Ş.E., Küreli G., Dalkara T. (2022). Contractile Apparatus in CNS Capillary Pericytes. Neurophotonics.

[82] Rungta R.L., Chaigneau E., Osmanski B.-F., Charpak S. (2018). Vascular Compartmentalization of Functional Hyperemia from the Synapse to the Pia. Neuron.

[83] Bonney S.K., Nielson C.D., Shih A.Y., Tuma R.F., Breslin J.W., Peirce-Cottler S., Murfee W.L. (2025). Mural Cells, Microvessels, and Alzheimer’s Disease. Handbook of Microcirculation.

[84] Sewell M., Fialova N., Montagne A. (2025). Unraveling the Transcriptomic Landscape of Brain Vascular Cells in Dementia: A Systematic Review. Alzheimers Dement..

[85] Guo X., Xia S., Ge T., Lin Y., Hu S., Wu H., Xie X., Zhang B., Zhang S., Zeng J. (2024). Atp13a5 Marker Reveals Pericyte Specification in the Mouse Central Nervous System. J. Neurosci..

[86] Kozberg M.G., Munting L.P., Hanlin L.H., Auger C.A., van den Berg M.L., Denis de Senneville B., Hirschler L., Warnking J.M., Barbier E.L., Farrar C.T. (2025). Vasomotion Loss Precedes Impaired Cerebrovascular Reactivity and Microbleeds in Cerebral Amyloid Angiopathy. Brain Commun..

[87] Aldea R., Weller R.O., Wilcock D.M., Carare R.O., Richardson G. (2019). Cerebrovascular Smooth Muscle Cells as the Drivers of Intramural Periarterial Drainage of the Brain. Front. Aging Neurosci..

[88] Uemura M.T., Maki T., Ihara M., Lee V.M.Y., Trojanowski J.Q. (2020). Brain Microvascular Pericytes in Vascular Cognitive Impairment and Dementia. Front. Aging Neurosci..

[89] Zenaro E., Piacentino G., Constantin G. (2017). The Blood-Brain Barrier in Alzheimer’s Disease. Neurobiol. Dis..

[90] Zlokovic B.V., Deane R., Sallstrom J., Chow N., Miano J.M. (2005). Neurovascular Pathways and Alzheimer Amyloid Beta-Peptide. Brain Pathol..

[91] Nelson A.R., Sweeney M.D., Sagare A.P., Zlokovic B.V. (2016). Neurovascular Dysfunction and Neurodegeneration in Dementia and Alzheimer’s Disease. Biochim. Biophys. Acta.

[92] Wang Q., Huang X., Su Y., Yin G., Wang S., Yu B., Li H., Qi J., Chen H., Zeng W. (2022). Activation of Wnt/β-Catenin Pathway Mitigates Blood-Brain Barrier Dysfunction in Alzheimer’s Disease. Brain.

[93] Montagne A., Barnes S.R., Sweeney M.D., Halliday M.R., Sagare A.P., Zhao Z., Toga A.W., Jacobs R.E., Liu C.Y., Amezcua L. (2015). Blood-Brain Barrier Breakdown in the Aging Human Hippocampus. Neuron.

[94] Nikolakopoulou A.M., Montagne A., Kisler K., Dai Z., Wang Y., Huuskonen M.T., Sagare A.P., Lazic D., Sweeney M.D., Kong P. (2019). Pericyte Loss Leads to Circulatory Failure and Pleiotrophin Depletion Causing Neuron Loss. Nat. Neurosci..

[95] Cipolla M.J. (2009). The Cerebral Circulation.

[96] Girouard H., Iadecola C. (2006). Neurovascular Coupling in the Normal Brain and in Hypertension, Stroke, and Alzheimer Disease. J. Appl. Physiol..

[97] Chen Y., He Y., Han J., Wei W., Chen F. (2023). Blood-Brain Barrier Dysfunction and Alzheimer’s Disease: Associations, Pathogenic Mechanisms, and Therapeutic Potential. Front. Aging Neurosci..

[98] Hernandes M.S., Xu Q., Griendling K.K. (2022). Role of NADPH Oxidases in Blood–Brain Barrier Disruption and Ischemic Stroke. Antioxidants.

[99] Kalsoom U.-E., Jin J., Meng W., Su Y., Wu C., Wu Q. (2026). The Role of NADPH Oxidases in Central Nervous System Regulation of Hypertension: Mechanisms and Therapeutic Insights. Biomed. Pharmacother..

[100] Watson A.N., Berthiaume A.-A., Faino A.V., McDowell K.P., Bhat N.R., Hartmann D.A., Shih A.Y. (2020). Mild Pericyte Deficiency Is Associated with Aberrant Brain Microvascular Flow in Aged PDGFRβ+/- Mice. J. Cereb. Blood Flow. Metab..

[101] Li Y., Wei W., Wang R.K. (2018). Capillary Flow Homogenization during Functional Activation Revealed by Optical Coherence Tomography Angiography Based Capillary Velocimetry. Sci. Rep..

[102] Li H., Guo Q., Inoue T., Polito V.A., Tabuchi K., Hammer R.E., Pautler R.G., Taffet G.E., Zheng H. (2014). Vascular and Parenchymal Amyloid Pathology in an Alzheimer Disease Knock-in Mouse Model: Interplay with Cerebral Blood Flow. Mol. Neurodegener..

[103] Garthwaite J. (2008). Concepts of Neural Nitric Oxide-Mediated Transmission. Eur. J. Neurosci..

[104] Chagnot A., Garcia D.J., McQuaid C., Cholewa-Waclaw J., McDade K., Dando O., Wardlaw J.M., Smith C., Montagne A. (2026). Abrupt Pericyte Loss Precedes Endothelial Activation in Cerebral Small Vessel Disease. BioRxiv.

[105] Stefancova D., Chagnot A., Sewell M., Fialova N., McQuaid C., Uweru O.J., Becker S., Romero-Bernal M., Mungall W., Walczak-Gillies V. (2026). Pericyte Loss Reprogrammes Capillary Endothelium and Drives White Matter Injury in Small Vessel Disease. bioRxiv.

[106] Procter T.V., Williams A., Montagne A. (2021). Interplay between Brain Pericytes and Endothelial Cells in Dementia. Am. J. Pathol..

[107] Zhu W., Huang H., Yang S., Luo X., Zhu W., Xu S., Meng Q., Zuo C., Zhao K., Liu H. (2019). Dysfunctional Architecture Underlies White Matter Hyperintensities with and without Cognitive Impairment. J. Alzheimers Dis..

[108] Nasrabady S.E., Rizvi B., Goldman J.E., Brickman A.M. (2018). White Matter Changes in Alzheimer’s Disease: A Focus on Myelin and Oligodendrocytes. Acta Neuropathol. Commun..

[109] Schager B., Brown C.E. (2020). Susceptibility to Capillary Plugging Can Predict Brain Region Specific Vessel Loss with Aging. J. Cereb. Blood Flow. Metab..

[110] Lahna D., Schwartz D.L., Woltjer R., Black S.E., Roese N., Dodge H., Boespflug E.L., Keith J., Gao F., Ramirez J. (2022). Venous Collagenosis as Pathogenesis of White Matter Hyperintensity. Ann. Neurol..

[111] Selkoe D.J., Hardy J. (2016). The Amyloid Hypothesis of Alzheimer’s Disease at 25 Years. EMBO Mol. Med..

[112] Attems J. (2005). Sporadic Cerebral Amyloid Angiopathy: Pathology, Clinical Implications, and Possible Pathomechanisms. Acta Neuropathol..

[113] van Veluw S.J., Benveniste H., Bakker E.N.T.P., Carare R.O., Greenberg S.M., Iliff J.J., Lorthois S., Van Nostrand W.E., Petzold G.C., Shih A.Y. (2024). Is CAA a Perivascular Brain Clearance Disease? A Discussion of the Evidence to Date and Outlook for Future Studies. Cell Mol. Life Sci..

[114] Keable A., Fenna K., Yuen H.M., Johnston D.A., Smyth N.R., Smith C., Al-Shahi Salman R., Samarasekera N., Nicoll J.A.R., Attems J. (2016). Deposition of Amyloid β in the Walls of Human Leptomeningeal Arteries in Relation to Perivascular Drainage Pathways in Cerebral Amyloid Angiopathy. Biochim. Biophys. Acta.

[115] van Veluw S.J., Hou S.S., Calvo-Rodriguez M., Arbel-Ornath M., Snyder A.C., Frosch M.P., Greenberg S.M., Bacskai B.J. (2020). Vasomotion as a Driving Force for Paravascular Clearance in the Awake Mouse Brain. Neuron.

[116] Berthiaume A.-A., Schmid F., Stamenkovic S., Coelho-Santos V., Nielson C.D., Weber B., Majesky M.W., Shih A.Y. (2022). Pericyte Remodeling Is Deficient in the Aged Brain and Contributes to Impaired Capillary Flow and Structure. Nat. Commun..

[117] Nortley R., Korte N., Izquierdo P., Hirunpattarasilp C., Mishra A., Jaunmuktane Z., Kyrargyri V., Pfeiffer T., Khennouf L., Madry C. (2019). Amyloid β Oligomers Constrict Human Capillaries in Alzheimer’s Disease via Signaling to Pericytes. Science.

[118] Dai W., Lopez O.L., Carmichael O.T., Becker J.T., Kuller L.H., Gach H.M. (2009). Mild Cognitive Impairment and Alzheimer Disease: Patterns of Altered Cerebral Blood Flow at MR Imaging. Radiology.

[119] Korte N., Nortley R., Attwell D. (2020). Cerebral Blood Flow Decrease as an Early Pathological Mechanism in Alzheimer’s Disease. Acta Neuropathol..

[120] Che P., Cai L., Liu F., Wang Y., Zhang Y., Wang Y., Lu Q., Piao Z., Zhang X., Qin W. (2025). Cerebral Perfusion Is Correlated with Cerebral Metabolism and Amyloid Deposition in Alzheimer’s Disease. Transl. Psychiatry.

[121] Wang L., Du Y., Wang K., Xu G., Luo S., He G. (2016). Chronic Cerebral Hypoperfusion Induces Memory Deficits and Facilitates Aβ Generation in C57BL/6J Mice. Exp. Neurol..

[122] Hattori Y., Enmi J.-I., Iguchi S., Saito S., Yamamoto Y., Tsuji M., Nagatsuka K., Kalaria R.N., Iida H., Ihara M. (2016). Gradual Carotid Artery Stenosis in Mice Closely Replicates Hypoperfusive Vascular Dementia in Humans. J. Am. Heart Assoc..

[123] Kaloss A.M., Browning J.L., Li J., Pan Y., Watsen S., Sontheimer H., Theus M.H., Olsen M.L. (2025). Meningeal Vascular Aβ Deposition Associates with Cerebral Hypoperfusion and Compensatory Collateral Remodeling. Alzheimer’s Res. Ther..

[124] Steinman J., Sun H.-S., Feng Z.-P. (2021). Microvascular Alterations in Alzheimer’s Disease. Front. Cell Neurosci..

[125] Colin M., Dujardin S., Schraen-Maschke S., Meno-Tetang G., Duyckaerts C., Courade J.-P., Buée L. (2020). From the Prion-like Propagation Hypothesis to Therapeutic Strategies of Anti-Tau Immunotherapy. Acta Neuropathol..

[126] Merlini M., Wanner D., Nitsch R.M. (2016). Tau Pathology-Dependent Remodelling of Cerebral Arteries Precedes Alzheimer’s Disease-Related Microvascular Cerebral Amyloid Angiopathy. Acta Neuropathol..

[127] Mawuenyega K.G., Sigurdson W., Ovod V., Munsell L., Kasten T., Morris J.C., Yarasheski K.E., Bateman R.J. (2010). Decreased Clearance of CNS Beta-Amyloid in Alzheimer’s Disease. Science.

[128] Carare R.O., Bernardes-Silva M., Newman T.A., Page A.M., Nicoll J.a.R., Perry V.H., Weller R.O. (2008). Solutes, but Not Cells, Drain from the Brain Parenchyma along Basement Membranes of Capillaries and Arteries: Significance for Cerebral Amyloid Angiopathy and Neuroimmunology. Neuropathol. Appl. Neurobiol..

[129] Iliff J.J., Wang M., Liao Y., Plogg B.A., Peng W., Gundersen G.A., Benveniste H., Vates G.E., Deane R., Goldman S.A. (2012). A Paravascular Pathway Facilitates CSF Flow through the Brain Parenchyma and the Clearance of Interstitial Solutes, Including Amyloid β. Sci. Transl. Med..

[130] Iliff J.J., Wang M., Zeppenfeld D.M., Venkataraman A., Plog B.A., Liao Y., Deane R., Nedergaard M. (2013). Cerebral Arterial Pulsation Drives Paravascular CSF-Interstitial Fluid Exchange in the Murine Brain. J. Neurosci..

[131] Wardlaw J.M., Benveniste H., Nedergaard M., Zlokovic B.V., Mestre H., Lee H., Doubal F.N., Brown R., Ramirez J., MacIntosh B.J. (2020). Perivascular Spaces in the Brain: Anatomy, Physiology and Pathology. Nat. Rev. Neurol..

[132] Hartz A.M.S., Miller D.S., Bauer B. (2010). Restoring Blood-Brain Barrier P-Glycoprotein Reduces Brain Amyloid-Beta in a Mouse Model of Alzheimer’s Disease. Mol. Pharmacol..

[133] Storck S.E., Meister S., Nahrath J., Meißner J.N., Schubert N., Di Spiezio A., Baches S., Vandenbroucke R.E., Bouter Y., Prikulis I. (2016). Endothelial LRP1 Transports Amyloid-β(1-42) across the Blood-Brain Barrier. J. Clin. Invest..

[134] Hong S., Beja-Glasser V.F., Nfonoyim B.M., Frouin A., Li S., Ramakrishnan S., Merry K.M., Shi Q., Rosenthal A., Barres B.A. (2016). Complement and Microglia Mediate Early Synapse Loss in Alzheimer Mouse Models. Science.

[135] Pan R.-Y., He L., Zhang J., Liu X., Liao Y., Gao J., Liao Y., Yan Y., Li Q., Zhou X. (2022). Positive Feedback Regulation of Microglial Glucose Metabolism by Histone H4 Lysine 12 Lactylation in Alzheimer’s Disease. Cell Metab..

[136] Christie R., Yamada M., Moskowitz M., Hyman B. (2001). Structural and Functional Disruption of Vascular Smooth Muscle Cells in a Transgenic Mouse Model of Amyloid Angiopathy. Am. J. Pathol..

[137] Niwa K., Younkin L., Ebeling C., Turner S.K., Westaway D., Younkin S., Ashe K.H., Carlson G.A., Iadecola C. (2000). Abeta 1-40-Related Reduction in Functional Hyperemia in Mouse Neocortex during Somatosensory Activation. Proc. Natl. Acad. Sci. USA.

[138] Shin H.K., Jones P.B., Garcia-Alloza M., Borrelli L., Greenberg S.M., Bacskai B.J., Frosch M.P., Hyman B.T., Moskowitz M.A., Ayata C. (2007). Age-Dependent Cerebrovascular Dysfunction in a Transgenic Mouse Model of Cerebral Amyloid Angiopathy. Brain.

[139] Sagare A., Deane R., Bell R.D., Johnson B., Hamm K., Pendu R., Marky A., Lenting P.J., Wu Z., Zarcone T. (2007). Clearance of Amyloid-Beta by Circulating Lipoprotein Receptors. Nat. Med..

[140] Yang J., Wise L., Fukuchi K.-I. (2020). TLR4 Cross-Talk with NLRP3 Inflammasome and Complement Signaling Pathways in Alzheimer’s Disease. Front. Immunol..

[141] Baik S.H., Kang S., Lee W., Choi H., Chung S., Kim J.-I., Mook-Jung I. (2019). A Breakdown in Metabolic Reprogramming Causes Microglia Dysfunction in Alzheimer’s Disease. Cell Metab..

[142] Colonna M., Butovsky O. (2017). Microglia Function in the Central Nervous System During Health and Neurodegeneration. Annu. Rev. Immunol..

[143] Escartin C., Galea E., Lakatos A., O’Callaghan J.P., Petzold G.C., Serrano-Pozo A., Steinhäuser C., Volterra A., Carmignoto G., Agarwal A. (2021). Reactive Astrocyte Nomenclature, Definitions, and Future Directions. Nat. Neurosci..

[144] Zhong R., Xu Y., Williams J.W., Li L. (2024). Loss of TREM2 Diminishes CAA despite an Overall Increase of Amyloid Load in Tg-SwDI Mice. Alzheimers Dement..

[145] Heneka M.T., Kummer M.P., Stutz A., Delekate A., Schwartz S., Vieira-Saecker A., Griep A., Axt D., Remus A., Tzeng T.-C. (2013). NLRP3 Is Activated in Alzheimer’s Disease and Contributes to Pathology in APP/PS1 Mice. Nature.

[146] Mi Y., Qi G., Vitali F., Shang Y., Raikes A.C., Wang T., Jin Y., Brinton R.D., Gu H., Yin F. (2023). Loss of Fatty Acid Degradation by Astrocytic Mitochondria Triggers Neuroinflammation and Neurodegeneration. Nat. Metab..

[147] Zeppenfeld D.M., Simon M., Haswell J.D., D’Abreo D., Murchison C., Quinn J.F., Grafe M.R., Woltjer R.L., Kaye J., Iliff J.J. (2017). Association of Perivascular Localization of Aquaporin-4 with Cognition and Alzheimer Disease in Aging Brains. JAMA Neurol..

[148] Simon M., Wang M.X., Ismail O., Braun M., Schindler A.G., Reemmer J., Wang Z., Haveliwala M.A., O’Boyle R.P., Han W.Y. (2022). Loss of Perivascular Aquaporin-4 Localization Impairs Glymphatic Exchange and Promotes Amyloid β Plaque Formation in Mice. Alzheimers Res. Ther..

[149] Liddelow S.A., Guttenplan K.A., Clarke L.E., Bennett F.C., Bohlen C.J., Schirmer L., Bennett M.L., Münch A.E., Chung W.-S., Peterson T.C. (2017). Neurotoxic Reactive Astrocytes Are Induced by Activated Microglia. Nature.

[150] Long X., Yao X., Jiang Q., Yang Y., He X., Tian W., Zhao K., Zhang H. (2020). Astrocyte-Derived Exosomes Enriched with miR-873a-5p Inhibit Neuroinflammation via Microglia Phenotype Modulation after Traumatic Brain Injury. J. Neuroinflammation.

[151] Liao K., Niu F., Hu G., Yang L., Dallon B., Villarreal D., Buch S. (2020). Morphine-Mediated Release of miR-138 in Astrocyte-Derived Extracellular Vesicles Promotes Microglial Activation. J. Extracell. Vesicles.

[152] González-Molina L.A., Villar-Vesga J., Henao-Restrepo J., Villegas A., Lopera F., Cardona-Gómez G.P., Posada-Duque R. (2021). Extracellular Vesicles from 3xTg-AD Mouse and Alzheimer’s Disease Patient Astrocytes Impair Neuroglial and Vascular Components. Front. Aging Neurosci..

[153] Armulik A., Genové G., Mäe M., Nisancioglu M.H., Wallgard E., Niaudet C., He L., Norlin J., Lindblom P., Strittmatter K. (2010). Pericytes Regulate the Blood–Brain Barrier. Nature.

[154] Daneman R., Zhou L., Kebede A.A., Barres B.A. (2010). Pericytes Are Required for Blood–Brain Barrier Integrity during Embryogenesis. Nature.

[155] Smyth L.C.D., Highet B., Jansson D., Wu J., Rustenhoven J., Aalderink M., Tan A., Li S., Johnson R., Coppieters N. (2022). Characterisation of PDGF-BB:PDGFRβ Signalling Pathways in Human Brain Pericytes: Evidence of Disruption in Alzheimer’s Disease. Commun. Biol..

[156] van Assema D.M.E., Lubberink M., Bauer M., van der Flier W.M., Schuit R.C., Windhorst A.D., Comans E.F.I., Hoetjes N.J., Tolboom N., Langer O. (2012). Blood-Brain Barrier P-Glycoprotein Function in Alzheimer’s Disease. Brain.

[157] Deo A.K., Borson S., Link J.M., Domino K., Eary J.F., Ke B., Richards T.L., Mankoff D.A., Minoshima S., O’Sullivan F. (2014). Activity of P-Glycoprotein, a β-Amyloid Transporter at the Blood-Brain Barrier, Is Compromised in Patients with Mild Alzheimer Disease. J. Nucl. Med..

[158] Wallace A., Zhou O., Hoang M.-N., Porter G., Padilla C., Marshall C. (2026). Lecanemab and Amyloid-Related Imaging Abnormalities: Real-World Data from a Single Center Experience. Alzheimer’s Dement. Diagn. Assess. Dis. Monit..

[159] van Etten E.S., Mahinrad S., Grill J.D., Salloway S., Atri A., Cogswell P.M., Benzinger T.L.S., Iwatsubo T., Iadecola C., Lemere C.A. (2026). Amyloid-Related Imaging Abnormalities (ARIA) in Anti-Amyloid Therapies for Alzheimer’s Disease: An Update from the Alzheimer’s Association ARIA Workgroup. Alzheimers Dement..

[160] Boche D., Zotova E., Weller R.O., Love S., Neal J.W., Pickering R.M., Wilkinson D., Holmes C., Nicoll J.a.R. (2008). Consequence of Abeta Immunization on the Vasculature of Human Alzheimer’s Disease Brain. Brain.

[161] Solopova E., Romero-Fernandez W., Harmsen H., Ventura-Antunes L., Wang E., Shostak A., Maldonado J., Donahue M.J., Schultz D., Coyne T.M. (2023). Fatal Iatrogenic Cerebral β-Amyloid-Related Arteritis in a Woman Treated with Lecanemab for Alzheimer’s Disease. Nat. Commun..

[162] Provensi G., Carta F., Nocentini A., Supuran C.T., Casamenti F., Passani M.B., Fossati S. (2019). A New Kid on the Block? Carbonic Anhydrases as Possible New Targets in Alzheimer’s Disease. Int. J. Mol. Sci..

[163] Solesio M.E., Peixoto P.M., Debure L., Madamba S.M., de Leon M.J., Wisniewski T., Pavlov E.V., Fossati S. (2018). Carbonic Anhydrase Inhibition Selectively Prevents Amyloid β Neurovascular Mitochondrial Toxicity. Aging Cell.

[164] Maki T., Okamoto Y., Carare R.O., Hase Y., Hattori Y., Hawkes C.A., Saito S., Yamamoto Y., Terasaki Y., Ishibashi-Ueda H. (2014). Phosphodiesterase III Inhibitor Promotes Drainage of Cerebrovascular β-Amyloid. Ann. Clin. Transl. Neurol..

[165] Blair G.W., Janssen E., Stringer M.S., Thrippleton M.J., Chappell F., Shi Y., Hamilton I., Flaherty K., Appleton J.P., Doubal F.N. (2022). Effects of Cilostazol and Isosorbide Mononitrate on Cerebral Hemodynamics in the LACI-1 Randomized Controlled Trial. Stroke.

[166] Bath P.M., Mhlanga I., Woodhouse L.J., Doubal F., Oatey K., Montgomery A.A., Wardlaw J.M. (2023). LACI-2 Trial Investigators Cilostazol and Isosorbide Mononitrate for the Prevention of Progression of Cerebral Small Vessel Disease: Baseline Data and Statistical Analysis Plan for the Lacunar Intervention Trial-2 (LACI-2) (ISRCTN14911850). Stroke Vasc. Neurol..

[167] Wardlaw J.M., Woodhouse L.J., Mhlanga I.I., Oatey K., Heye A.K., Bamford J., Cvoro V., Doubal F.N., England T., Hassan A. (2023). Isosorbide Mononitrate and Cilostazol Treatment in Patients with Symptomatic Cerebral Small Vessel Disease: The Lacunar Intervention Trial-2 (LACI-2) Randomized Clinical Trial. JAMA Neurol..

[168] Alarcon-Martinez L., Yilmaz-Ozcan S., Yemisci M., Schallek J., Kılıç K., Can A., Di Polo A., Dalkara T. (2018). Capillary Pericytes Express α-Smooth Muscle Actin, Which Requires Prevention of Filamentous-Actin Depolymerization for Detection. eLife.

[169] Yang A.C., Vest R.T., Kern F., Lee D.P., Agam M., Maat C.A., Losada P.M., Chen M.B., Schaum N., Khoury N. (2022). A Human Brain Vascular Atlas Reveals Diverse Mediators of Alzheimer’s Risk. Nature.

[170] Nebel R.A., Aggarwal N.T., Barnes L.L., Gallagher A., Goldstein J.M., Kantarci K., Mallampalli M.P., Mormino E.C., Scott L., Yu W.H. (2018). Understanding the Impact of Sex and Gender in Alzheimer’s Disease: A Call to Action. Alzheimers Dement..

[171] Neu S.C., Pa J., Kukull W., Beekly D., Kuzma A., Gangadharan P., Wang L.-S., Romero K., Arneric S.P., Redolfi A. (2017). Apolipoprotein E Genotype and Sex Risk Factors for Alzheimer Disease: A Meta-Analysis. JAMA Neurol..

[172] Buckley R.F., Mormino E.C., Rabin J.S., Hohman T.J., Landau S., Hanseeuw B.J., Jacobs H.I.L., Papp K.V., Amariglio R.E., Properzi M.J. (2019). Sex Differences in the Association of Global Amyloid and Regional Tau Deposition Measured by Positron Emission Tomography in Clinically Normal Older Adults. JAMA Neurol..

